# A coherent approach for analysis of the Illumina HumanMethylation450 BeadChip improves data quality and performance in epigenome-wide association studies

**DOI:** 10.1186/s13059-015-0600-x

**Published:** 2015-02-15

**Authors:** Benjamin Lehne, Alexander W Drong, Marie Loh, Weihua Zhang, William R Scott, Sian-Tsung Tan, Uzma Afzal, James Scott, Marjo-Riitta Jarvelin, Paul Elliott, Mark I McCarthy, Jaspal S Kooner, John C Chambers

**Affiliations:** Department of Epidemiology and Biostatistics, Imperial College London, London, W2 1PG UK; Wellcome Trust Centre for Human Genetics, University of Oxford, Oxford, UK; Institute of Health Sciences, University of Oulu, P.O. Box 5000, Oulu, FI-90014 Finland; Ealing Hospital NHS Trust, Middlesex, UB1 3HW UK; National Heart and Lung Institute, Imperial College London, London, W12 0NN UK; Biocenter Oulu, University of Oulu, P.O. Box 5000, Aapistie 5A, Oulu, FI-90014 Finland; Unit of Primary Care, Oulu University Hospital, Kajaanintie 50, P.O. Box 20, FI-90220 Oulu, 90029 OYS Finland; Department of Children and Young People and Families, National Institute for Health and Welfare, Aapistie 1, Box 310, Oulu, FI-90101 Finland; MRC-PHE Centre for Environment and Health, School of Public Health, Imperial College London, London, W2 1PG UK; Oxford Centre for Diabetes Endocrinology and Metabolism, University of Oxford, Oxford, UK; Imperial College Healthcare NHS Trust, London, W12 0HS UK

## Abstract

DNA methylation plays a fundamental role in the regulation of the genome, but the optimal strategy for analysis of genome-wide DNA methylation data remains to be determined. We developed a comprehensive analysis pipeline for epigenome-wide association studies (EWAS) using the Illumina Infinium HumanMethylation450 BeadChip, based on 2,687 individuals, with 36 samples measured in duplicate. We propose new approaches to quality control, data normalisation and batch correction through control-probe adjustment and establish a null hypothesis for EWAS using permutation testing. Our analysis pipeline outperforms existing approaches, enabling accurate identification of methylation quantitative trait loci for hypothesis driven follow-up experiments.

**Electronic supplementary material:**

The online version of this article (doi:10.1186/s13059-015-0600-x) contains supplementary material, which is available to authorized users.

## Background

DNA methylation is involved in the regulation of numerous biological processes, including gene expression [1], cell differentiation [2] and X-chromosome inactivation [3]. Altered DNA methylation has been linked to complex human diseases including cancer [4], schizophrenia [5], multiple sclerosis [6] and type 2 diabetes [7-9]. Recent technological developments, in particular the release of the Illumina Infinium HumanMethylation450 BeadChip (450 K methylation array), make it possible to measure DNA methylation on a genome-wide scale [10]. However, the 450 K methylation array includes multiple different probe types, each using different chemistry. Furthermore the methylation assay involves bisulphite conversion of DNA and other steps that introduce assay variability and batch effects. Multiple methods have been proposed for analysis of the complex data generated by the 450 K methylation array [11-17]; however, there is currently no consensus on the optimal analysis pipeline.

We propose a comprehensive approach to the analysis of 450 K methylation array data. Our method was developed using data from over 2,600 samples from the London Life Sciences Prospective Population (LOLIPOP) study, including 36 samples measured in duplicate and identifies differential methylation on a single-marker level. Our pipeline, termed CPACOR (incorporating Control Probe Adjustment and reduction of global CORrelation), performs superiorly to published methods, and provides a blueprint for the analysis of large-scale Epigenome-Wide Association Studies (EWAS).

## Results and discussion

### Initial quantification and quality control

We analysed two DNA methylation datasets: a population study of type 2 diabetes comprising 2,687 samples; and a technical replication dataset comprising 36 samples measured in duplicate **(**Materials and Methods**)**. To maximise the impact of technical factors in the replication dataset, the initial and repeat sample analyses were carried out in separate batches.

We performed an initial top-level quality control following analysis recommendations given by Illumina. We excluded 22 samples (sample call rate <98% or incorrect gender). The distributions for methylation values differ between autosomal and gender chromosome markers (Additional file 1: Figure S1); we therefore analyse these separately. Markers that are predicted to cross-hybridise [18], with a SNP in the probe-sequence, or that measure methylation at non-CpG sites were retained but flagged.

### Evaluating the detection *P* value threshold

**Figure 1:**
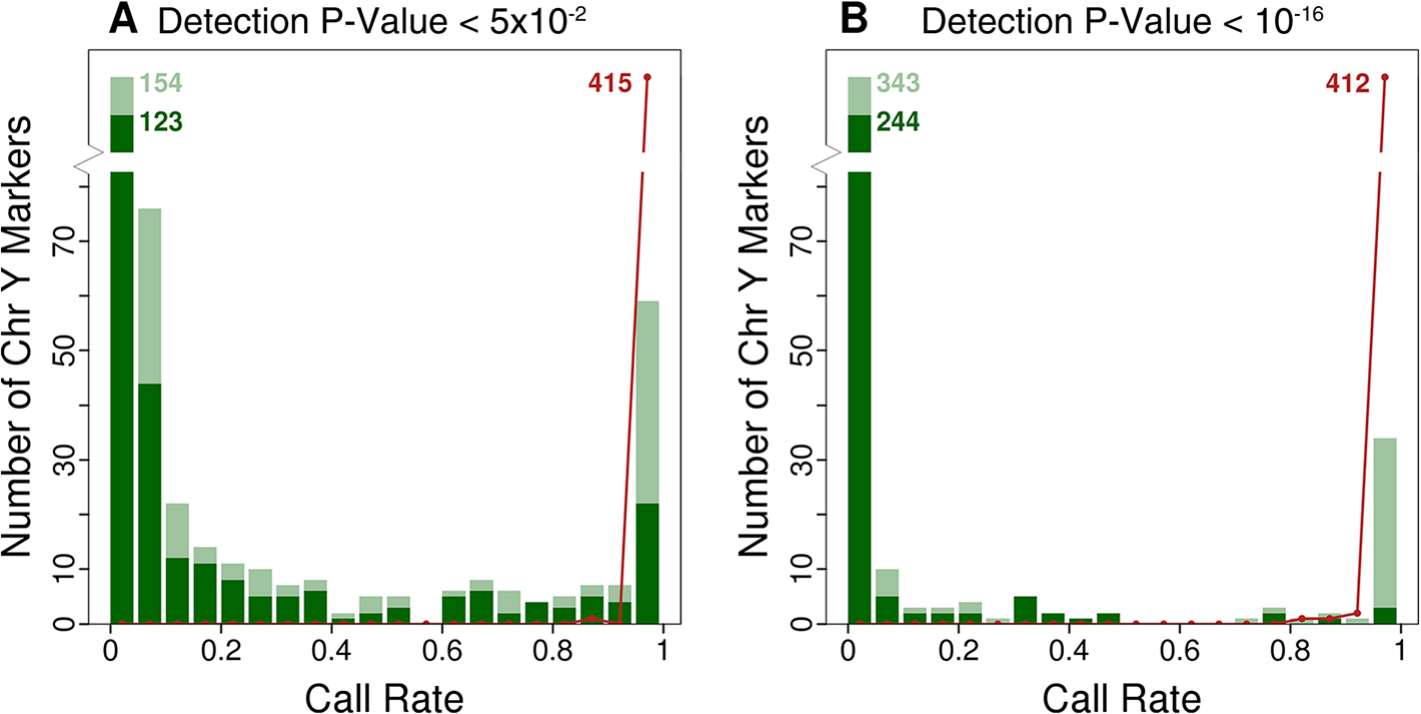
**Marker call rates on the Y-chromosome.** Distribution of call rates for 416 Y-chromosome markers in males (red points and red line) and females (green bars). Y-chromosome markers in females are represented in light green if their respective probes sequences are predicted to cross-hybridise with multiple genomic regions. Values greater than 80 are represented by numbers. **(A)** For a detection threshold *P* <0.05 more than 50% of Y-chromosome markers show non-zero call rates (call rate >0.05%) in females, even though females do not possess a Y chromosome. **(B)** For a detection threshold *P* <10^−16^ only 10% of Y-chromosome markers show non-zero call rates in females. Marker call rates in males (shown in red) are not materially affected by the more stringent detection *P* value threshold.

We initially used a detection *P* value of *P* <0.05 for marker calling based on Illumina recommendations. We noted though that calculated detection *P* values reported by minfi [15] range from 1 to 2.2 × 10^−16^, with values lower than 2.2 × 10^−16^ reported as zero (Additional file 1: Figure S2). To investigate the impact of detection *P* value threshold, we first evaluated call rates on the Y-chromosome among females in the population study; these are expected to be zero for all 416 markers. In contrast, we found that >50% of Y-chromosome markers had non-zero call rates in females (Figure 1), suggesting that the default detection *P* value (*P* <0.05) is not sufficient to prevent spurious results. When the detection *P* value threshold is lowered to *P* <10^−16^ the proportion of Y-chromosome markers with non-zero call rate in females is reduced from 55% to 10%. The majority of these remaining markers represent previously unidentified cross-hybridising probes (Additional file 1: Table S1). A more stringent detection threshold does not impact materially on Y-chromosome calling in males (Figure 1 and Additional file 1: Figure S3).

To extend these findings to autosomal markers, we quantified the proportion of extreme values (outliers) at each marker in the population study as a metric for quality of marker calling (Methods). Adoption of a more stringent detection *P* value threshold (*P* <10^−16^) reduces the proportion of outlying values, especially at markers with lower call rates, consistent with improved calling (Additional file 1: Figure S4).

As a final test, we compared results for the 36 samples that were measured in duplicate. We observe a higher correlation (*P* = 2.91 × 10^−11^) between duplicate pairs when a detection *P* value threshold of *P* <10^−16^ is applied compared to a threshold *P* <0.05 (Additional file 1: Figure S5), providing further evidence for improved quantification of methylation with a more stringent detection *P* value threshold.

This approach provides a roadmap for researchers to determine the detection *P* value threshold that is optimal for their dataset. Based on our results, we chose *P* <10^−16^ as detection *P* value threshold, providing a high accuracy at minimal loss of data. We recalculated sample call rates and excluded one further sample from the population study dataset with a call rate below 98% leaving 2,664 samples for further analysis.

### Data normalisation

Data normalisation is frequently applied in the analysis of microarray data to reduce technical biases across measurements. To establish a consensus approach for normalisation of the 450 K methylation array we assessed the performance of 10 different normalisation methods [11,14,18-21] using the relationship between beta values for the 36 samples measured in duplicate (Additional file 1: Figure S6). The highest correlations between the paired measurements of methylation were achieved after quantile normalisation of intensity values for markers, subdivided by probe type, probe sub-type and colour channel (Additional file 1: Figure S7 and S8, Table S2). Functional normalisation (FN) [22], subset within array normalisation (SWAN) [20] and quantile normalisation of beta values performed significantly worse, while within-array approaches showed little or no improvement compared to non-normalised data.

While correlation between technical replicates assesses Type-I statistical error, it may not assess over-normalisation. To quantify the ability to detect true signal after each normalisation method, we performed spike-in simulations based on the population study. Case–control status was randomly assigned to samples and beta values of 100 randomly selected markers were increased (‘spiked’) in the case samples. We then determined the proportion of the spiked markers that were ranked in the top 100 methylation markers by univariate regression analysis. Confirming our initial finding, quantile normalisation of intensity values performs best, followed by quantile normalisation of beta values and subset quantile normalisation. Whereas most methods lead to improved performance, some over-normalise resulting in a reduction of true signal compared to no normalisation (Additional file 1: Figure S9; Table S3).

On the basis of these results, which are in agreement with previous findings [23,24], we performed quantile normalisation of intensity values for all samples in this study.

### Removal of technical biases

**Figure 2:**
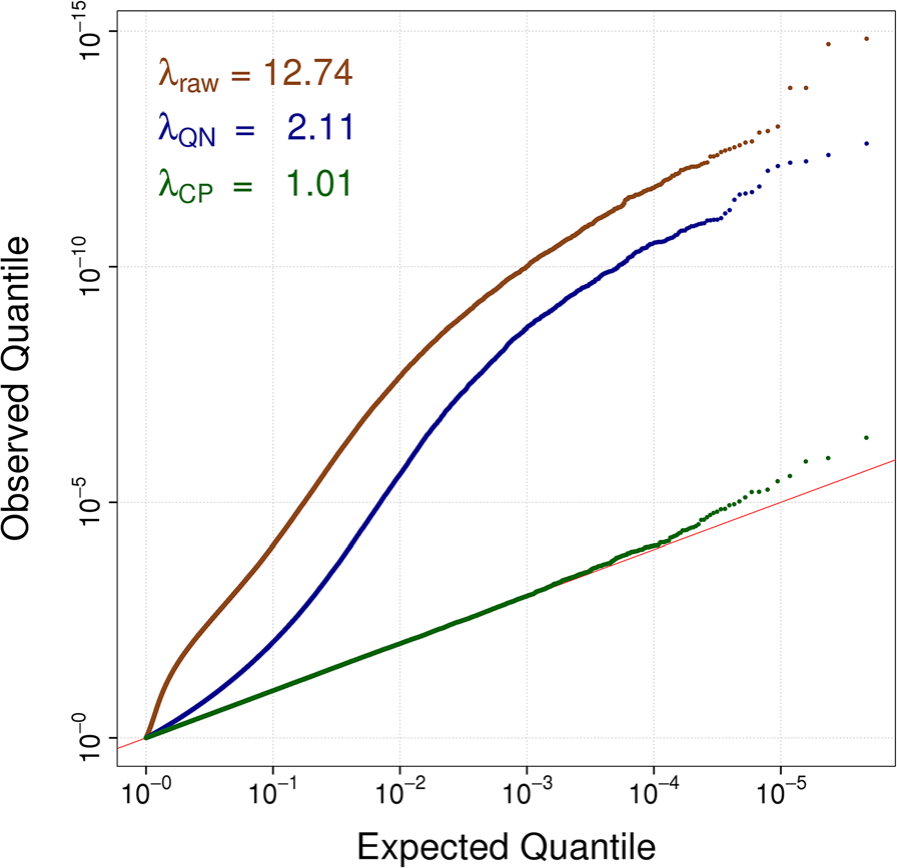
**Correcting for statistical inflation due to technical biases.** Quantile-Quantile (QQ) plot for the comparison of 36 samples measured in duplicate reveals high statistical inflation before (Genomic Inflation Factor λ_raw_ = 12.74; brown points) and after quantile normalisation (λ_QN_ = 2.11; green points) due to technical biases. Batch-correction based on control probes removes technical biases and statistical inflation (λ_CP_ = 1.01; green points).

To investigate whether there were remaining technical biases after quantile normalisation, we used linear regression to compare the paired measurements of beta values from the 36 samples measured in duplicate. We observed a high degree of statistical inflation (λ = 2.11, Figure 2) indicating strong residual biases between the duplicates, consistent with batch and other technical effects.

Existing methods to further reduce technical biases require knowledge of relevant experimental factors such as bisulfite conversion batch, array number, position on array, date or time [25]. These data may not be available, or where available may not accurately measure the technical bias (Additional file 1: Figure S10). To overcome these limitations and improve upon existing approaches, we developed Control Probe Adjustment as a new method to correct for technical biases in the 450 K methylation data. We first retrieved signal intensities for the 450 K methylation array control probes, which assess multiple aspects of the chemistry involved in quantification of methylation, such as bisulfite-conversion efficiency (Additional file 1: Table S4). To take into account the high degree of correlation between these control probes (Additional file 1: Figure S11), we performed a principal component analysis (PCA) of control probe intensities, and then included the principal components (PCs) as linear predictors in the regression analysis of the 36 samples measured in duplicate. The PCs correlated closely with multiple technical parameters, including bisulfite batch and plates (Additional file 1: Figure S12). Adjustment for the first 30 PCs almost entirely removed test statistic inflation consistent with effective correction for batch and technical effects (λ = 1.01; Figure 2, Additional file 1: Figure S13).

To further evaluate this strategy, we applied Control Probe Adjustment to the population study of 2,664 samples. This effectively removed the biases introduced by known technical factors (Additional file 1: Figure S14).

### Null hypothesis and global correlation patterns

**Figure 3:**
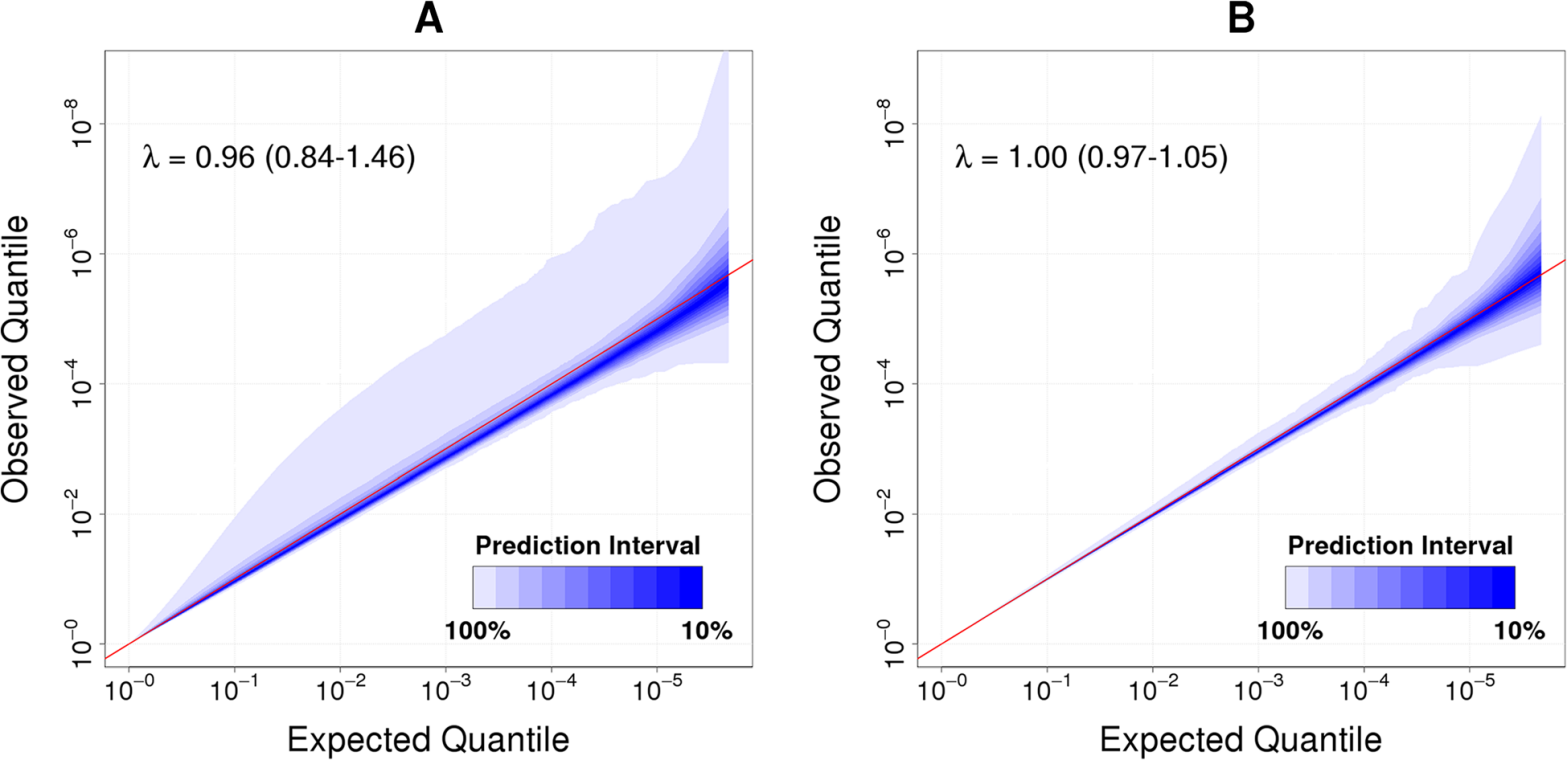
**Prediction interval under the null hypothesis of no association.** Quantile-quantile (QQ) prediction intervals for 1,000 permutations of the case–control status. The λ-value represents the median (2.5 percentile to 97.5 percentile) of all genomic inflation factors λ. **(A)** Quantile normalisation and adjustment for control probe PCs. Under no association we observe and overall statistical deflation, but also a high degree of statistical inflation for a small number of permutations. **(B)** Quantile normalisation, adjustment for control probe PCs, gender, age, white blood cells and PC1-5. Adjustments abolishes overall statistical deflation and results in a substantially more narrow prediction interval.

To determine the *P* value distribution under the null hypothesis we randomly re-assigned case–control status among the 2,664 samples of the population study and performed a logistic regression for each marker using quantile normalised beta values and adjusting for control probe PCs. We repeated this 1,000 times to give 1,000 sets of *P* values under no association. Despite permutation of the case–control status to remove true association we observed substantial departure from the null expectation. This includes both overall statistical deflation for the majority of permutations, but also a small number of permutations with a high degree of statistical inflation (λ_median_ = 0.96; λ_2.5%tile_ = 0.84; λ_97.5%tile_ = 1.46, Figure 3A).

Theoretically expected *P* values are based on the assumption of independence for each test. In contrast we observe a high degree of correlation (and anti-correlation) between 1,000 randomly chosen markers (Additional file 1: Figure S15). We hypothesised that this correlation between markers reduces the number of independent tests and may explain the apparent deflation of *P* values. To test this hypothesis, we randomly reassigned beta values for each marker to re-establish independence between markers. This effectively abolished test-statistics deflation and revealed a narrow prediction interval around the expected (λ_median_ = 1.00; λ_2.5%tile_ = 1.00; λ_97.5%tile_ = 1.01; Additional file 1: Figure S16).

### Factors driving global correlation patterns

**Figure 4:**
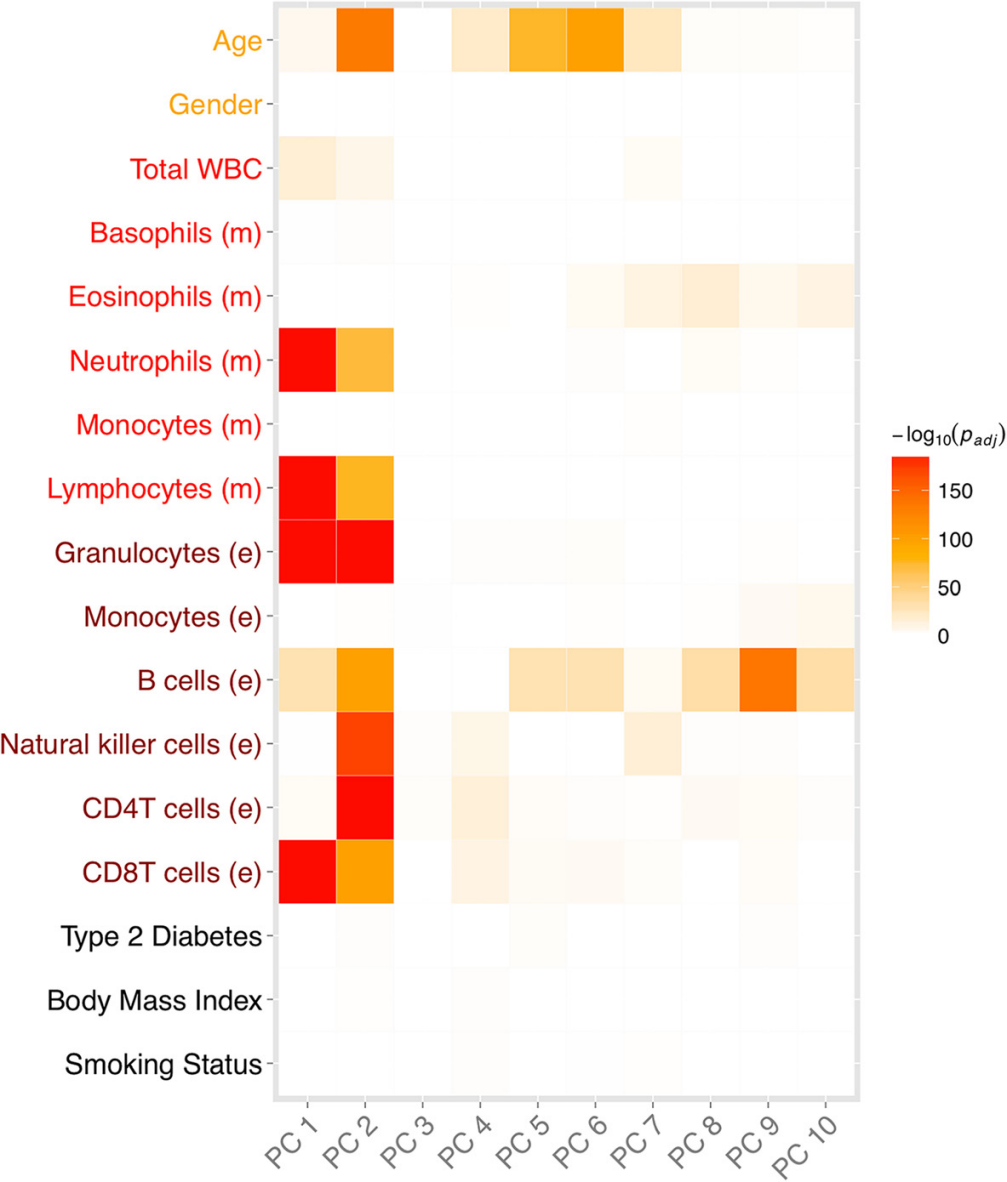
**Principal component analysis identifies global correlation patterns.** We carried out a PCA of the methylation data (based on residuals after quantile normalisation and Control Probe Adjustment) to identify the primary patterns of covariation and used the PCs to explore relationships to biological factors such as measured (m) and estimated (e) white blood-cell subsets, gender, age and others. Colours represent *P* values for correlation of different factors with PCs 1 to 10.

Correlation between methylation markers may arise from technical and biological confounders. We therefore carried out a further PCA of the population study dataset to identify the primary patterns of covariation between the genome-wide measurements of autosomal methylation in peripheral blood. We then used the PCs to explore relationships of methylation to technical and biological factors (Figure 4).

The first three PCs were strongly associated with multiple white blood cell sub-populations. To further explore this aspect we generated a complementary set of white blood cell subpopulations, which were estimated from the methylation data itself [26]. The estimated white blood cell subsets accurately reproduce white blood cell measurements (Pearson correlation coefficient r = 0.82-0.56; Additional file 1: Figure S17), but provide cell type proportions of four additional lymphocyte sub-populations. We also found significant correlations of PCs with age, but not with any other clinical variables.

Adjustment for biological factors in the population samples reduced the correlation between markers and test statistic inflation, with the greatest reduction resulting from adjustment for white blood cell subpopulations (Additional file 1: Figure S18-S19). To make a final correction for global covariation that is still unaccounted by the biological factors included in the regression we performed a final PCA of the residuals after adjustment for technical and biological factors. Adjustment for the first five PCs (which explain 3.7% of the variation; Additional file 1: Figure S20), further reduced the correlation between markers (λ_median_ = 1.00; λ_2.5%tile_ = 0.97; λ_97.5%tile_ = 1.05; Figure 3B). On the basis of these results we calculated a 95% prediction interval and propose an epigenome wide significance threshold of *P* <10^−7^ that is consistent with approximately 470,000 independent tests.

### Impact on local correlation

Previous studies have reported an increased degree of correlation between neighbouring CpG sites (<1 kb distance) [27,28], which are likely to reflect biologically functional units. We replicated these findings, and also show that our adjustments for technical and biological factors remove correlation between markers with a high genomic distance (>1 kb) while retaining correlation between markers in direct genomic neighbourhood (<1 kb) (Additional file 1: Figure S21, Table S5). These observations support the view that our approach to data analysis preferentially removes the long-range correlations between markers that are more likely to be spurious.

### Performance

**Figure 5:**
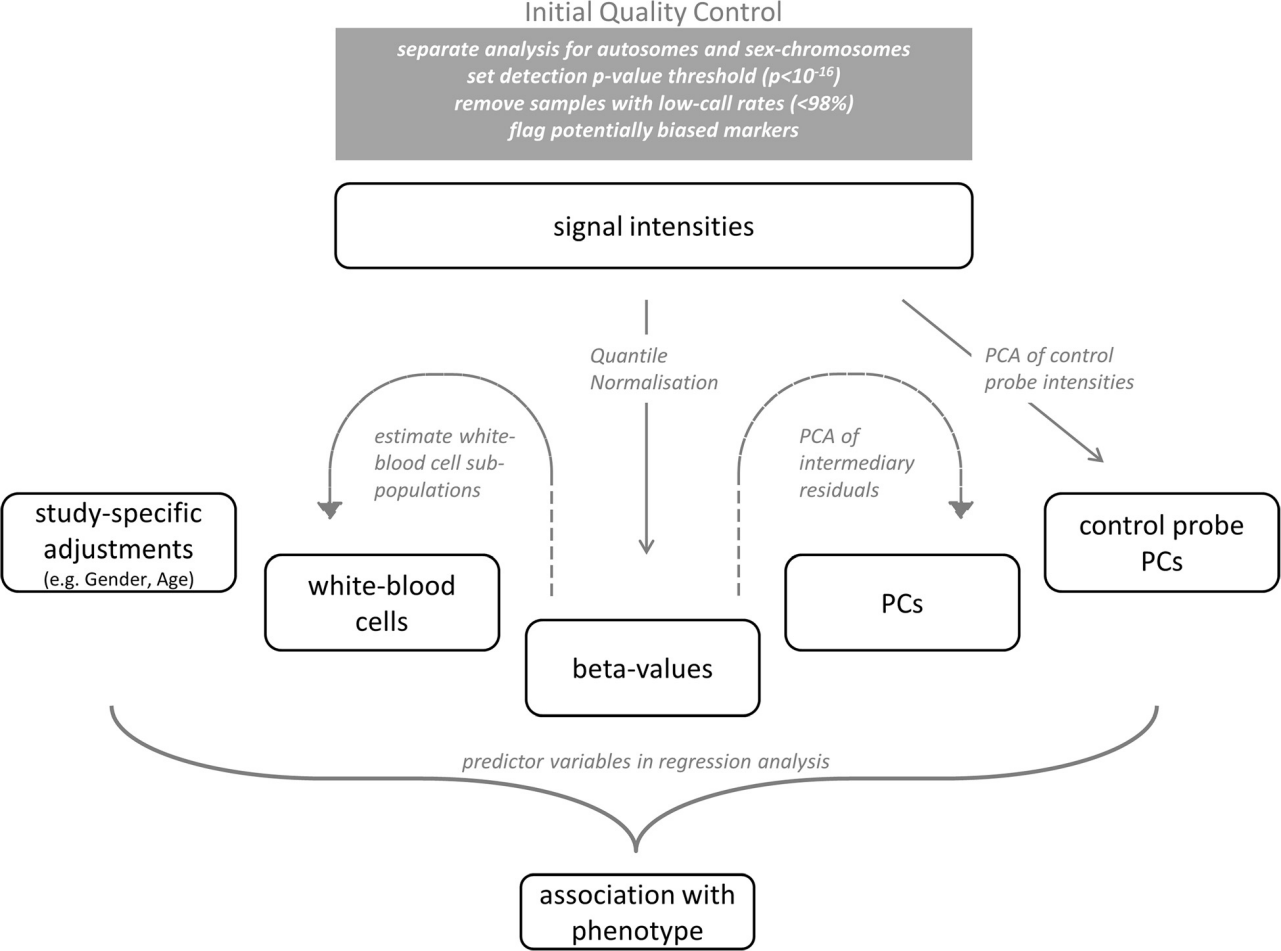
**Workflow of CPACOR (incorporating Control Probe Adjustment and reduction of global CORrelation) for Epigenome-wide Association Studies using the Illumina HumanMethylation450 BeadChip.** Signal intensities are filtered for multiple Quality Control criteria followed by quantile normalisation and calculation of beta values. In addition intensities from Illumina control probes are used to derive principal components (control-probe PCs). Based on beta values, proportions of white blood cell subpopulations are estimated and PCs are derived (from intermediary residuals; see Methods for details). To detect differential methylation regression analysis is performed for each methylation marker predicting disease status as a function of the (quantile normalised) beta value adjusted for technical and biological factors and PCs.

**Figure 6:**
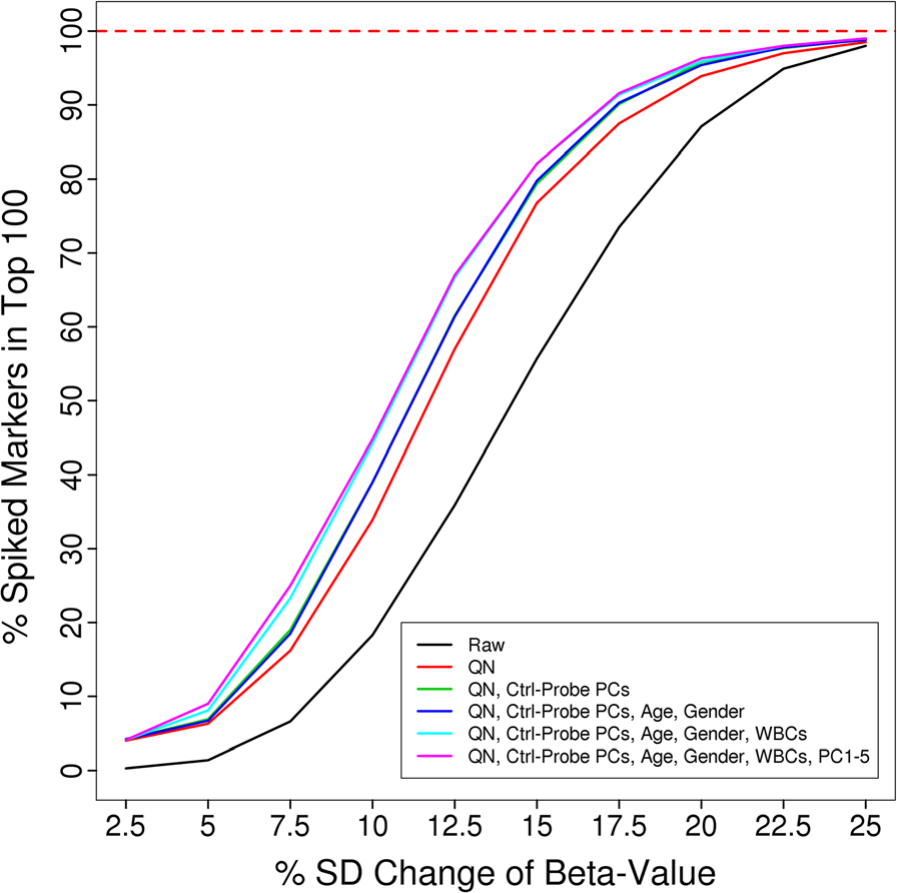
**Simulation analysis shows successive improvement of each stage of the CPACOR pipeline.** We increased (‘spiked’) beta values of 100 randomly selected markers and determined the proportion of the spiked markers that were ranked among the top 100. The proportion of spiked markers achieving top 100 ranks is improved successively by each of the stages of the CPACOR pipeline.

We used simulated case–control datasets to assess the performance of the CPACOR analysis pipeline (Figure 5, Additional file 1: Table S6). Based on the spike-in approach described above, we show that the proportion of spiked markers achieving high rank is improved successively by each of the stages of our pipeline including quantile normalisation, adjustment for control probes, and adjustment for biological factors (Figure 6, Additional file 1: Table S7). We conclude that these adjustments increase the power to identify true association signals and reduce systematic biases between samples.

We used simulations to compare the performance of our analysis pipeline with published methods [11-17]. This analysis focuses on single marker comparisons to identify differentially methylated CpG sites, rather than a multi-marker approach [29] to avoid regional biases introduced by the non-random selection of CpG sites targeted by the HumanMethylation450 BeadChip [10]. We found that most of the published methods could not be completed using datasets of >2,000 samples, even on a dedicated high-performance computing cluster with 2 TB of RAM (Additional file 1: Figure S22; Table S8). In contrast our approach achieves improved computational performance through parallelisation. Although different methylation studies may require different approaches to analysis, results from spiked data of a smaller dataset (250 cases, 250 controls) indicate that CPACOR performs significantly better than published methods (Additional file 1: Figure S22, Tables S9 and S10).

### Adjustment using reference-free approaches

Reduction of statistical inflation is crucial for the analysis of EWAS. Here we use direct adjustment for known biological confounders to achieve this. Several recently developed methods for epigenome-wide association attempt to adjust for biological confounders without prior knowledge or reference datasets [30,31]. These so-called ‘reference-free’ approaches attempt to correct for biological confounders by identifying clusters of covariation in the data and removing this covariation by adjustment. For example, the EWASher method [30] attempts to reduce statistical inflation by constructing a methylation similarity matrix based on CpGs most strongly associated with the endpoint. It includes this similarity matrix as the covariance component in a Linear Mixed Model (LMM) regression. However, because this adjustment is based on methylation values of the most strongly associated CpGs, this approach may remove variation attributable to the endpoint (Additional file 1: Figure S23).

RefReeEWAS, a different reference-free approach, excludes variation attributable to the endpoint before adjustment [31]. However, we find that it performs substantially less well than CPACOR (Additional file 1: Figure S24). This may partly be explained by the very considerable computational requirement which limit the number of bootstraps for deriving *P* values.

Our data suggest that reference-free approaches perform less well than the direct adjustment implemented in our pipeline. However, for tissue samples where relevant reference datasets are not available these approaches may provide a strategy to reduce statistical inflation.

### Marker subtypes and sex chromosomes

To investigate for potential biases arising from other marker specific properties, we assessed the impact of markers in three categories (Materials and methods): (1) non-CpG markers; (2) cross-hybridising markers; and (3) markers with a SNP in the probe-sequence (Additional file 1: Table S11). We found very little evidence to suggest these markers reduce overall data quality. Including them during quantile normalisation does not materially affect correlation between technical duplicates (mean r = 0.9979 in both cases). *P* value distributions under no association show no evidence that non-CpG markers or markers with SNPs in the probe sequence are more likely to generate spurious results, but we observe a slight increase in correlation for cross-hybridising markers (Additional file 1: Figure S25). We therefore recommend retaining, but flagging these markers.

Adjustment for technical and biological factors also reduces correlation between markers on the sex chromosomes, although to a lesser extent than autosomal markers, resulting in broader prediction intervals (Additional file 1: Figure S26). This suggests a higher probability of both Type-1 and Type-2 errors during analysis of sex-chromosome data, compared to autosomal results.

## Conclusions

The emergence of the Illumina 450 k methylation array now enables investigation of the relationships between DNA methylation and phenotype in population studies. We provide a blueprint for an EWAS analysis pipeline based on data from the Illumina 450 Methylation array. We show that the default detection *P* value is insufficiently stringent to prevent spurious results, identify the optimal approach to data normalisation, describe a new, highly effective method for dealing with technical bias, and demonstrate the importance of accounting for biological confounders. On the basis of these results we demonstrate an epigenome-wide significance threshold of *P* <10^−7^, that is consistent with Bonferroni correction. We show that our approach significantly outperforms existing methods for identification of true association. Furthermore our approach is scalable and, unlike many existing methods, capable of handling large-scale datasets involving several thousand samples. Our comprehensive set of instructions for the analysis of Illumina 450 k methylation will advance the ability of EWAS to accurately identify methylation quantitative trait loci for hypothesis driven follow-up experiments.

## Materials and methods

In the first section we describe in detail the consecutive steps of our EWAS analysis pipeline. The corresponding scripts are provided in Additional file 2 (usage requires knowledge of R-programming and scripts may have to be adapted to the user’s hardware and software requirements). The subsequent sections provide details on data generation and additional analyses performed to compare and evaluate the various methodological components.

### EWAS analysis pipeline

1. Quality control
  Illumina Infinium 450 K data are retrieved using the minfi R package (version 1.2.0) [15] and downstream analyses are performed using minfi and R. We remove 65 single nucleotide polymorphism (SNP) markers and apply Illumina Background Correction to all intensity values. Methylation markers on autosomes and gender chromosomes are analysed separately. A detection *P* value threshold of *P* <10^−16^ was chosen and intensity values with detection *P* ≥10^−16^ are set to missing data. We determine the proportion of missing data points per sample, enabling calculation of the sample call rate and exclude samples with sample call rate <98%. We also remove samples with swapped gender labels identified by high call rates for Y-chromosome markers.
2. Quantile normalisation of intensity values
  Intensity values are separated into six different probe-type categories defined by colour channel, probe-type and M/U subtype (Type-I M red, Type-I U red, Type-I M green, Type-I U green, Type-II red, Type-II green). Within each category intensity values are quantile normalised using limma [32]. Normalised intensity values are then used to calculate the percentage methylation at each CpG site (beta value).
3. Control Probe Adjustment
  We use intensity values from the Infinium 450 K control probes (Additional file 1: Table S4) to adjust for technical bias. Control probe intensities (excluding negative control probes) are obtained using minfi [15]. A PCA of the control probe intensities is performed and the resulting PCs 1 to 30 are subsequently included as linear predictors in regression models (steps 5 and 6).
4. Estimation of white blood cell sub-populations
  Six white blood cell sub-populations are estimated using the approach described by Houseman *et al.* [26]. Estimates are based on 500 markers most informative of white blood cell subpopulations as measured by the Illumina Infinium 27 K methylation array. Of these 470 are also present on the Illumina Infinium 450 K methylation array and are therefore used in this analysis. Estimated white blood cell subpopulations (WBCest) and (measured) total white blood cell counts (WBCtot) are subsequently included as linear predictors in regression models (steps 5 and 6).
5. PCA of intermediary residuals
  To make a final correction for global covariation that is still unaccounted for, we perform a linear regression predicting the (quantile normalised) beta values adjusted for technical and biological factors and study-specific confounders such as gender and age (1).

1 $$ Beta\ (QN) \sim age + gender + WB{C}_{est} + WB{C}_{tot} + PC1-{30}_{ctrl- probes} $$

We then perform a PCA on the resulting regression residuals (excluding markers with missing data) and include PC 1 to 5 as linear predictors in the final regression model (step 6).

6. Logistic regression analysis to identify differential methylation
  To detect differential methylation we perform a final (logistic) regression analysis for each methylation marker predicting disease status Y as a function of the beta value adjusted for technical and biological factors and PCs (2).

2 $$ Y \sim Beta\ (QN) + age + gender + WB{C}_{est} + WB{C}_{tot} + PC1-{30}_{ctrl- probes} + PC1-5 $$

### Data generation

Two DNA methylation datasets were generated in this study: (1) a population study of 2,687 samples (1,080 Type 2 Diabetes cases, 1,607 controls); and (2) a replication dataset of 36 samples measured in duplicate. Genomic DNA was extracted from peripheral blood and analysed in batches of 288 samples. To *maximise* the impact of technical factors in the replication dataset, the initial and repeat sample measurements were carried out in separate batches. Methylation was quantified following standard protocol (Infinium_HD_Methylation_Assay_Guide_15019519_B) with 1 ug of DNA as starting material and an elution volume of 14 uL after bisulphite conversion (using the EZ-96 methylation kit; Zymo). Microarrays were imaged using an Illumina HiScan scanner.

### Initial quality control

Illumina Infinium 450 K data were retrieved as described and an initial top-level Quality Control was performed following the analysis recommendations given by the array manufacturer. In brief, we applied Illumina Background Correction to all intensity values and calculate the percentage methylation at each CpG site assayed (the beta value). An initial detection *P* value threshold of *P* <0.05 was chosen based on Illumina recommendations; beta values with detection *P* ≥0.05 were set to missing data. We determined the proportion of missing data points per sample and per marker, enabling calculation of sample and marker call rates, respectively. For the population study we excluded 17 samples with sample call rate <98%. We also removed five samples with swapped gender labels identified by high call rates for Y-chromosome markers. After re-evaluation of the detection *P* value threshold, beta values with detection *P* ≥10^−16^ were set to missing data. We re-calculated sample call rates and excluded one further individual with sample call rate <98% from the study.

### Outliers and outlier rate

For each methylation marker we define outliers based on the interquartile range (IQR), such that beta values are considered as outliers if they fall below Quartile 1–1.5 × IQR or above Quartile 3 + 1.5 × IQR. Outlier rates are calculated as the number of outlying beta values divided by the number of non-missing beta values.

### Data normalisation

We evaluated 10 methods to data normalisation: (1) quantile normalisation of beta values separated by probe-type and colour channel (Type II, Type I red, Type I green) using limma [32]; (2) quantile normalisation of intensity values separated by colour channel (red and green channel; termed QN-I2) using limma [32]; (3) quantile normalisation of intensity values separated by colour channel and probe-type (Type I red, Type I green, Type II red, Type II green; termed QN-I4) using limma [32]; (4) quantile normalisation of intensity values separated by colour channel, probe type and M/U subtypes (Type I M red, Type I U red, Type I M green, Type I U green, Type II red, Type II green; termed QN-I6) using limma [32]; (5) Illumina Control Probe normalization as implemented by minfi [15] (‘*normalize.illumina.control*’; not to be confused with CPA); (6) subset within-array normalisation (SWAN) [20]; (7) peak-based correction [11]; (8) Beta MIxture Quantile dilation (BMIQ) [19]; (9) subset quantile normalisation [14]; and (10) functional normalisation (FN) [22]. All normalisation methods were implemented using the R packages supplied with the publications.

After each normalisation we determined the Pearson correlation coefficients between replicates for the 36 samples measured in duplicate. Pearson correlation coefficients are calculated (1) at the marker level: correlation coefficient between the 36 paired measurements for each of the 470,000 markers assayed (thus generating approximately 470,000 test results; Additional file 1: Figure S6A); and (2) at the sample level: correlation co-efficient between the paired measurements of the approximately 470,000 markers assayed in each of the 36 duplicate samples (thus generating 36 test results; Additional file 1: Figure S6B). A paired Wilcoxon test was used to assess the difference between the normalisation methods.

To assess the degree of true signal detectable after each normalisation method, we performed spike-in simulations. Based on the population study, disease labels were randomised to generate 10 permutated datasets. From each permuted dataset 100 markers were randomly selected and spiked. For each ‘spike-marker’ raw beta values of samples with a case label are increased by a defined proportion of the standard deviation of the respective marker. Based on these spiked beta values we calculate intensity values for the methylated and the unmethylated probe, such that for half of the spiked probes the methylated intensity is changed and for the other half the unmethylated intensity is changed. Negative intensity values resulting from this process are set to zero. Intensity values were spiked over a range of magnitudes (as percentage of SD of the beta value) resulting in 10 sets per magnitude (10 permutations per magnitude). Using univariate logistic regression we calculated *P* values for each permuted datasets and ranked the 100 spiked markers by their association *P* values. For each magnitude the 10 permuted dataset provide a total of 1,000 ranks for 1,000 spiked markers.

### Analysis of technical replicates

To assess the degree of technical biases and batch effects, we analysed a technical replication dataset comprising 36 samples measured in duplicate. To maximise the impact of technical factors the initial and repeat sample analyses were carried out in separate batches. We performed regression analyses to identify differentially methylated positions between replicates. Using paired linear regression we predict replicate status as a function of the beta value with and without adjustments for control probe PCs.

3 $$ Replicate \sim Beta\ (QN) $$

4 $$ Replicate \sim Beta\ (QN) + PC1-30 ctrl- probes $$

### Batch correction using ComBat

We performed batch correction for technical technical replication dataset based on quantile normalised beta values using ComBat [25] to compare its performance to CPA. ComBat, as implemented in ChAMP (*champ.runCombat*) [16], performs a batch correction based on the Bead-Chip (Sentrix ID) and returns corrected methylation values. All samples measured on relevant Bead-Chips were included for batch correction. To avoid ComBat deliberately preserving differences attributable to the outcome of interest (replicate status), gender was defined as the Sample Group.

### Permutations of the disease status

To make permutation-analysis of the large-scale population study computationally tractable for 1,000 permutations we performed a linear regression of model (5) and retrieved the residuals. These were used as predictors in a logistic regression, with the (permuted) disease-status (Y_perm_) as outcome (6). This approach is in almost perfect agreement with a conventional model that directly adjusts for all linear predictors (2): we calculated coefficients of determination (R^2^-values) for -log(*P* values) and beta-coefficients with respect to results from model (2) and found R^2^ > 0.999 (analysis performed for permutation 1 to 10).

5 $$ Beta\ (QN) \sim age + gender + WB{C}_{est} + WB{C}_{tot} + PC1-{30}_{ctrl- probes} + PC1-5 $$

6 $$ {Y}_{perm} \sim residuals $$

### Assessment of white blood cell estimates

For the population study estimated white blood cell subpopulations (WBC_est_) explain a higher proportion of variance in the methylation data than measured white blood cell subpopulations, which may reflect the wider range of lymphocyte sub-classes in estimated sub-populations. Adjustment for white blood cells is therefore based on estimated white blood cell sub-populations and (measured) total white blood cell counts (WBC_tot_).

### Analysis of global correlation patterns (heatmap)

To identify global correlation patterns that can be explained by biological factors, we performed a PCA based on methylation residuals after quantile normalisation (QN) and CPA (7). PCs were linked to multiple phenotypes (age, gender, white blood cells, and so on) using linear regression. *P* values of association (between the PCs and the phenotypes) were Bonferroni-corrected and plotted on the -log_10_ scale (Figure 4).

7 $$ Beta\ (QN) \sim PC1-30 ctrl- probes $$

### Local correlation

Local correlation was determined for all possible pairs of autosomal markers up to 5,000 bp apart. Distance between markers was based on the annotated position of the CpG sites on the forward strand. Pearson correlation coefficient between marker pairs were calculated based on beta values (raw) and residuals derived from models (5) and (7). A large proportion of methylation markers show very little variation, which limits their ability to yield high correlation coefficients. To reflect the effect of genomic distance on correlation more appropriately we therefore selected the 5% most variable markers (based on raw beta values) and represent their correlation graphically on a continuous scale using a sliding 300 bp mean. To demonstrate that adjustments preferentially reduced correlation between markers with greater distance we calculated the difference in correlation coefficients per basepair distance (between two different adjustments). To determine statistical significance we then performed a linear regression of the differences and the genomic distance.

### Performance

Spike-in simulations were carried out as described. Each permuted dataset was then analysed using different stages of the CPACOR pipeline and other published 450 k analysis pipelines [11-17] (where computationally tractable). Only approaches providing a complete analysis pipeline from signal intensities to detection of differentially methylated CpG sites were considered. For each pipeline default parameters were chosen as specified and ranks were calculated as described.

### Reference-free approaches

Using ‘spike-in’ data generated as described, we assessed the performance of EWASher [30] and RefFreeEWAS [31]. Beta values were quantile normalised and control-probe PCs 1 to 30 were provided as covariates to adjust for technical biases. Because neither approach was computationally tractable for the complete dataset (2,664 samples), analysis was performed on a smaller dataset (250 cases, 250 controls).

EWASher was applied to all CpGs (including constitutively methylated CpGs). Default parameters were chosen as specified and results of each analysis step (linear regression, linear mixed model regression, linear mixed model regression + PCs) were retrieved.

Adjusted and unadjusted beta coefficients were calculated using RefFreeEWAS. Dimensionality was estimated as described by Houseman *et al.* (d = 133) and default parameters were chosen as specified. To derive *P* values we performed 50 bootstraps, which required 50 hours of compute time and 130 GB RAM.

### Marker categories

We assessed the following probe types for their impact on association test results:

1. Non-CpG markers. Autosomal probes that measure methylation at CpA and CpT rather than CpG sites (N = 2,995) based on the Illumina annotation.
2. Cross-mapping probes. Methylation probe sequences reported to map to >1 genomic location (N = 39,963) identified by Price *et al.* [18].
3. Probes with SNPs. Methylation markers with one or more SNPs located within the probe sequence (including the G-base of the CpG site) that have minor allele frequency >1% in the samples studied. (N = 75,702).

### Sex chromosomes

Analysis of methylation markers on the sex chromosomes was performed as described for autosomal markers, but separately in males (chromosome X and Y) and females (chromosome X). In addition to samples with autosomal call rates <98% we excluded samples with chromosome X and Y call rates <98%. This results in 1,780 samples for chromosome Y (25 samples excluded), 1,802 for chromosome X in males (3 samples excluded) and 859 samples for chromosome X in females. Separately for each dataset we performed quantile normalisation and adjusted for control probe PCs, age, white blood cells and PC 1 to 5.

### Data availability

Methylation array data can be accessed through the Gene Expression Omnibus at http://www.ncbi.nlm.nih.gov/geo/query/acc.cgi?acc=GSE55763.

## Additional files

Additional file 1:
**Contains all supplementary figures and tables.**
Additional file 2:
**Contains scripts and documentation for the CPACOR EWAS analysis pipeline.** Files can be read using a standard text-editor. Usage requires knowledge of R-programming and may have to be adapted to accommodate the software and hardware requirements specific to the user’s system.

## Abbreviations

CpA: Cytosine-phophate-Adenine site
CPACOR: Incorporating control probe adjustment and reduction of global CORrelation
CpG: Cytosine-phophate-Guanine site
CpT: Cytosine-phosphate-Thymine site
EWAS: Epigenome-wide association study
IQR: Interquartile range
PC: Principal component
PCA: Prinicipal component analysis
QN: Quantile normalisation
SNP: Single nucleotide polymorphism
WBC: White blood cells
