# Erratum to: A coherent approach for analysis of the Illumina HumanMethylation450 BeadChip improves data quality and performance in epigenome-wide association studies

**DOI:** 10.1186/s13059-016-0934-z

**Published:** 2016-04-21

**Authors:** Benjamin Lehne, Alexander W. Drong, Marie Loh, Weihua Zhang, William R. Scott, Sian-Tsung Tan, Uzma Afzal, Reiner Schulz, James Scott, Marjo-Ritta Jarvelin, Paul Elliott, Mark I. McCarthy, Jaspal S. Kooner, John C. Chambers

**Affiliations:** Department of Epidemiology and Biostatistics, Imperial College London, London, W2 1PG UK; Wellcome Trust Centre for Human Genetics, University of Oxford, Oxford, UK; Institute of Health Sciences, University of Oulu, P.O.Box 5000, Oulu, FI-90014 Finland; Ealing Hospital NHS Trust, Middlesex, UB1 3HW UK; National Heart and Lung Institute, Imperial College London, London, W12 0NN UK; Division of Genetics and Molecular Medicine, King’s College London, London, UK; Biocenter Oulu, University of Oulu, P.O.Box 5000, Aapistie 5A, Oulu, FI-90014 Finland; Unit of Primary Care, Oulu University Hospital, Kajaanintie 50, P.O.Box 20, FI-90220, Oulu, 90029 OYS Finland; Department of Children and Young People and Families, National Institute for Health and Welfare, Aapistie 1, Box 310, FI-90101 Oulu, Finland; Oxford Centre for Diabetes Endocrinology and Metabolism, University of Oxford, Oxford, UK; Imperial College Healthcare NHS Trust, London, W12 0HS UK

After the publication of this work [1] it was noticed that an author was omitted from the author list and author contributions. Please see below updated author contributions.
