# Supplementary material for: A coherent approach for analysis of the Illumina HumanMethylation450 BeadChip improves data quality and performance in epigenome-wide association studies
Source: Genome Biol. 2015 Feb 15;16(1):37. doi: 10.1186/s13059-015-0600-x (PMC4365767; doi:10.1186/s13059-015-0600-x)

Online Supplementary Material

A coherent approach for analysis of the Illumina HumanMethylation450 BeadChip improves data quality and performance in epigenome-wide association studies

Lehne and Drong *et al.*

Supplementary Table 1. Y-chromosome markers with non-zero call-rates in females (detection threshold $p<10^{-16}$) are predominantly caused by previously unidentified cross-hybridising probes (22 of 27 probes cross-hybridise). We first used BLAT to map the target sequence of Y-chromosome probes to the hg19 reference genome. To further take into account the effect of bisulphite conversion, we also mapped the probe sequence to the bisulphite-converted reference genomes (hg19, 4 strands) allowing for up to two mismatches, 90% sequence identity and no gaps.

Y-chromosome Marker	Reference genome		Bisulphite converted genome		
	Chr X	Autosomes	Chr X	Autosomes	**Total**
cg01086462	1	0	4	0	5
cg01209756	0	0	0	4	4
cg01707559	0	0	2	4	6
cg02002345	0	0	2	0	2
cg02233183	1	0	2	6	9
cg03244189	0	0	1	7	8
cg03278611	0	0	2	0	2
cg03359666	0	13	0	28	41
cg04170994	0	0	0	2	2
cg04689676	0	0	0	5	5
cg04792227	0	0	0	6	6
cg04964672	1	0	4	7	12
cg05618150	1	0	2	0	3
cg05678960	0	0	0	1	1
cg05782707	0	0	0	0	0
cg09197443	1	0	3	6	10
cg09223632	0	0	0	4	4
cg10422744	1	0	4	0	5
cg10835413	0	0	0	0	0
cg13851368	0	0	0	5	5
cg14180491	0	0	0	0	0
cg16552926	0	0	0	0	0
cg23308414	0	0	0	4	4
cg25012987	0	1	0	1	2
cg25032547	0	0	0	1	1
cg25059696	0	0	0	0	0
cg25363292	0	0	0	3	3

Supplementary Table 2. Correlations between 36 samples measured in duplicate, using different approaches to data-normalisation. Correlation coefficients are calculated A) at the sample level to derive a correlation co-efficient between the paired measurements of the ~470,000 markers assayed in each of the 36 duplicate samples and B) at the marker level to derive a correlation coefficient between the 36 paired measurements for each of the ~470,000 markers assayed. Highest correlations are seen for quantile normalisation on intensities. P values are for the comparison between approaches (pairwise Wilcoxon-test; two-tailed) of per sample results (blue) or per marker results (green).

	**Mean Correlations (SD)**		**Between method comparisons (P values)**										
**Normalisation**	**Per-sample**	**Per-marker**	**Subset QN**	**Raw**	**BMIQ**	**PBC**	**Illumina**	**FN**	**SWAN**	**QN-I2**	**QN-B**	**QN-I4**	**QN-I6**
**Subset QN**	0.9971 (0.0006)	0.3901 (0.3691)	-	<2.2e-308	<2.2e-308	<2.2e-308	4.6E-17	<2.2e-308	8.3E-68	1.9E-70	4.9E-03	1.0E-81	1.4E-122
**Raw**	0.9969 (0.0011)	0.3182 (0.3612)	8.2E-01	-	<2.2e-308	<2.2e-308	<2.2e-308	<2.2e-308	<2.2e-308	<2.2e-308	<2.2e-308	<2.2e-308	<2.2e-308
**BMIQ**	0.9970 (0.0011)	0.3224 (0.3519)	6.0E-01	1.3E-05	-	3.8E-111	<2.2e-308	<2.2e-308	<2.2e-308	<2.2e-308	<2.2e-308	<2.2e-308	<2.2e-308
**PBC**	0.9970 (0.0011)	0.3221 (0.3537)	5.3E-01	1.7E-07	2.0E-01	-	<2.2e-308	<2.2e-308	<2.2e-308	<2.2e-308	<2.2e-308	<2.2e-308	<2.2e-308
**Illumina**	0.9973 (0.0007)	0.3962 (0.3932)	3.8E-07	1.6E-02	2.8E-01	3.8E-01	-	4.3E-188	<2.2e-308	5.6E-19	8.7E-09	1.4E-23	4.9E-45
**FN**	0.9975 (0.0007)	0.3725 (0.3853)	1.4E-07	1.0E-06	2.9E-03	6.7E-03	4.9E-07	-	4.2E-05	<2.2e-308	7.4E-131	<2.2e-308	<2.2e-308
**SWAN**	0.9976 (0.0006)	0.3754 (0.3814)	4.3E-08	2.9E-11	2.6E-09	2.6E-09	2.1E-06	8.2E-03	-	7.2E-261	7.3E-86	2.0E-279	<2.2e-308
**QN-I2**	0.9978 (0.0006)	0.4039 (0.3696)	2.9E-11	5.8E-11	1.9E-08	2.6E-08	2.9E-11	8.9E-09	9.0E-03	-	<2.2e-308	<2.2e-308	<2.2e-308
**QN-B**	0.9979 (0.0004)	0.3922 (0.3687)	2.9E-11	5.8E-11	7.4E-09	1.1E-08	2.9E-11	7.3E-10	4.9E-03	3.5E-01	-	<2.2e-308	<2.2e-308
**QN-I4**	0.9978 (0.0006)	0.4049 (0.3685)	2.9E-11	5.8E-11	6.0E-09	6.0E-09	2.9E-11	4.9E-09	6.7E-04	4.6E-03	9.2E-01	-	<2.2e-308
**QN-I6**	0.9979 (0.0004)	0.4082 (0.3667)	2.9E-11	2.9E-11	2.9E-10	2.9E-10	2.9E-11	7.3E-10	7.4E-06	6.0E-06	1.9E-01	2.2E-03	-

Supplementary Table 3. Simulations show quantile normalisation of intensity values outperforms other normalisation methods (paired Wilcoxon rank test).

	% of spiked markers in top 100			improvement over previous method (P-value)
	Mean	1st Quartile	3rd Quartile	
SWAN	38.8%	7.0%	68.3%	-
Illumina	47.0%	7.8%	84.3%	2.83E-10
Raw	47.2%	4.8%	88.0%	9.81E-01
BMIQ	47.4%	7.0%	89.0%	3.11E-02
PBC	48.9%	10.0%	88.3%	5.11E-01
FN	50.1%	11.5%	87.0%	8.20E-02
Subset-QN	54.8%	13.0%	92.0%	4.19E-13
QN-B	55.8%	14.0%	93.0%	2.07E-08
QN-I2	56.7%	15.0%	94.3%	8.96E-03
QN-I4	56.8%	15.0%	95.0%	7.02E-01
QN-I6	57.1%	15.8%	94.0%	2.29E-03

Supplementary Table 4. Control Probes on the Illumina 450K methylation array.

Category	Description	No. of Types	No. of Probes
Bisulfite Conversion	Methylation at a site known to be methylated	3	10
Normalisation	Randomly permutated bisulphite-converted sequences containing no CpGs; Determines system background	4	186
Staining	Efficiency and sensitivity of staining step	2	2
Extension	Extension efficiency of A, T, C, and G nucleotides from a hairpin probe	4	4
Hybridisation	Hybridisation efficiency using synthetic targets instead of amplified DNA	3	3
Target Removal	Efficiency of stripping step after extension reaction	1	2
Specificity	Methylation at non-polymorphic T sites	3	9
Non-polymorphic	Methylation at a base in a non-polymorphic region of the genome	4	4

Supplementary Table 5. Correlation between methylation markers in genomic proximity before and after adjustment for technical and biological factors. QN: quantile normalisation; CPA: control probe adjustment.

Distance (bp)	N	beta-value (raw)		QN+CPA		Final model	
		Mean	SE	Mean	SE	Mean	SE
**1 - 500**	11126	0.768	0.002	0.775	0.002	0.744	0.002
**501 – 1000**	2587	0.575	0.006	0.555	0.007	0.520	0.007
**1001 – 1500**	1320	0.437	0.009	0.396	0.010	0.356	0.010
**1501 – 2000**	986	0.324	0.010	0.267	0.011	0.240	0.011
**2001 – 2500**	765	0.285	0.011	0.215	0.012	0.196	0.012
**2501 - 3000**	702	0.225	0.012	0.146	0.013	0.135	0.013
**3001 - 3500**	599	0.208	0.014	0.135	0.016	0.119	0.015
**3501 - 4000**	612	0.258	0.013	0.183	0.014	0.173	0.014
**4001 - 4500**	556	0.202	0.013	0.124	0.014	0.096	0.014
**4501 - 5000**	594	0.190	0.013	0.110	0.014	0.082	0.013

Supplementary Table 6. Analysis steps and software packages employed in the CPACOR analysis pipeline.

Analysis Step	R function and package
Extraction of signal intensities	read.450k(); minfi package
Background Subtraction	bgcorrect.illumina(); minfi package
Retrieval of detection P-values	detectionP(); minfi package
Principal Component Analysis (PCA)	prcomp()
Quantile Normalisation	normalizeQuantiles(); Limma package
Estimation of white-blood cell sub-populations	wbcInference-V110.R by Houseman *et al.*
Regression analysis	glm()

Supplementary Table 7. Simulations show significant performance improvements for each stage of the analysis pipeline (paired Wilcoxon rank test).

	% of spiked markers in top 100			Improvement over previous adjustment (P-value)
	Mean	1st Quartile	3rd Quartile	
no adjustment	47.2%	4.8%	88.0%	-
QN	57.1%	15.8%	94.0%	2.30E-14
+ Control Probe PCs	59.3%	19.0%	95.3%	2.46E-10
+ Gender, Age	59.2%	18.0%	95.3%	0.36.
+ WBC_{tot} + WBC_{est}	61.3%	24.0%	96.0%	6.75E-11
+ PC 1-5	61.7%	25.8%	96.0%	0.01

Supplementary Table 8. Existing pipelines for the analysis of Illumina 450K methylation array data.

Name	Pre-processing	Normalisation	Adjustments	Differential Methylation Analysis	Computational Requirements	Comment
Illumina Genome Studio	background subtraction, detection p<0.05	mean adjustment; normalization to internal controls	none specified	illumina custom model	only runs on MS windows	not scalable or usable with computing clusters.
Minfi	illumina control normalization; background subtraction	SWAN	none specified	linear regression for continuous, F-test for categorical	1.9 TB RAM, 1.5 hours	
IMA	peak corretion; detection $p<10^{-5}$, sample call-rate>75%	quantile normalisation (based on?)	none specified	linear regression (limma)	computationally not tractable for >2,600 samples	
Lumi	color bias; background correction	quantile normalisation (M+U)	none specified	linear regression (limma)	computationally not tractable for >2,600 samples	
Champ	SVD (visual)	BMIQ + ComBat	none specified	linear regression (limma); lasso DMR hunter	computationally not tractable for >2,600 samples	
CPACOR	background subtraction, detection $p<10^{-16}$, sample call-rate>98%	quantile normalisation (6 categories)	control probe PCs, gender, age, white-blood cells, PCs	logistic/linear regression	<150 GB RAM; <2hrs (on multicore cluster)	suitable for large-scale EWAS

Supplementary Table 9. Simulations show significant performance improvements of CPACOR over published analysis pipeline where these successfully completed analysis of the complete dataset (2,664 samples; paired Wilcoxon rank test).

	% of spiked markers in top 100			improvement over previous pipeline (P-value)
	Mean	1st Quartile	3rd Quartile	
Minfi	57.1%	20.8%	91.3%	-
CPACOR	61.7%	25.8%	96.0%	9.69E-13

Supplementary Table 10. Simulations show significant performance improvements of CPACOR over published analysis pipeline for analysis of a smaller dataset (500 samples; paired Wilcoxon rank test).

	% of spiked markers in top 100			improvement over previous pipeline (P-value)
	Mean	1st Quartile	3rd Quartile	
Minfi	49.6%	9.0%	88.0%	-
lumi	49.8%	10.0%	87.0%	2.54E-01
IMA	50.3%	7.8%	91.9%	3.76E-02
CHAMP	50.5%	6.5%	91.1%	8.36E-18
CPACOR	55.3%	12.5%	93.3%	2.00E-39

Supplementary Table 11. Methylation markers with specific properties

Marker property	N
Autosomal	473,961
X-chromosome	11,135
Y-chromosome	416
SNP under probe (autosomal)	64,672
Cross-hybridising (autosomal)	39,963
Non-CpGs (autosomal)	2,994

Supplementary Figure 1. Distribution of beta-values for autosomal and sex-chromosome markers (Red: overall; Green: Type 1 probes; Blue: Type 2 probes)

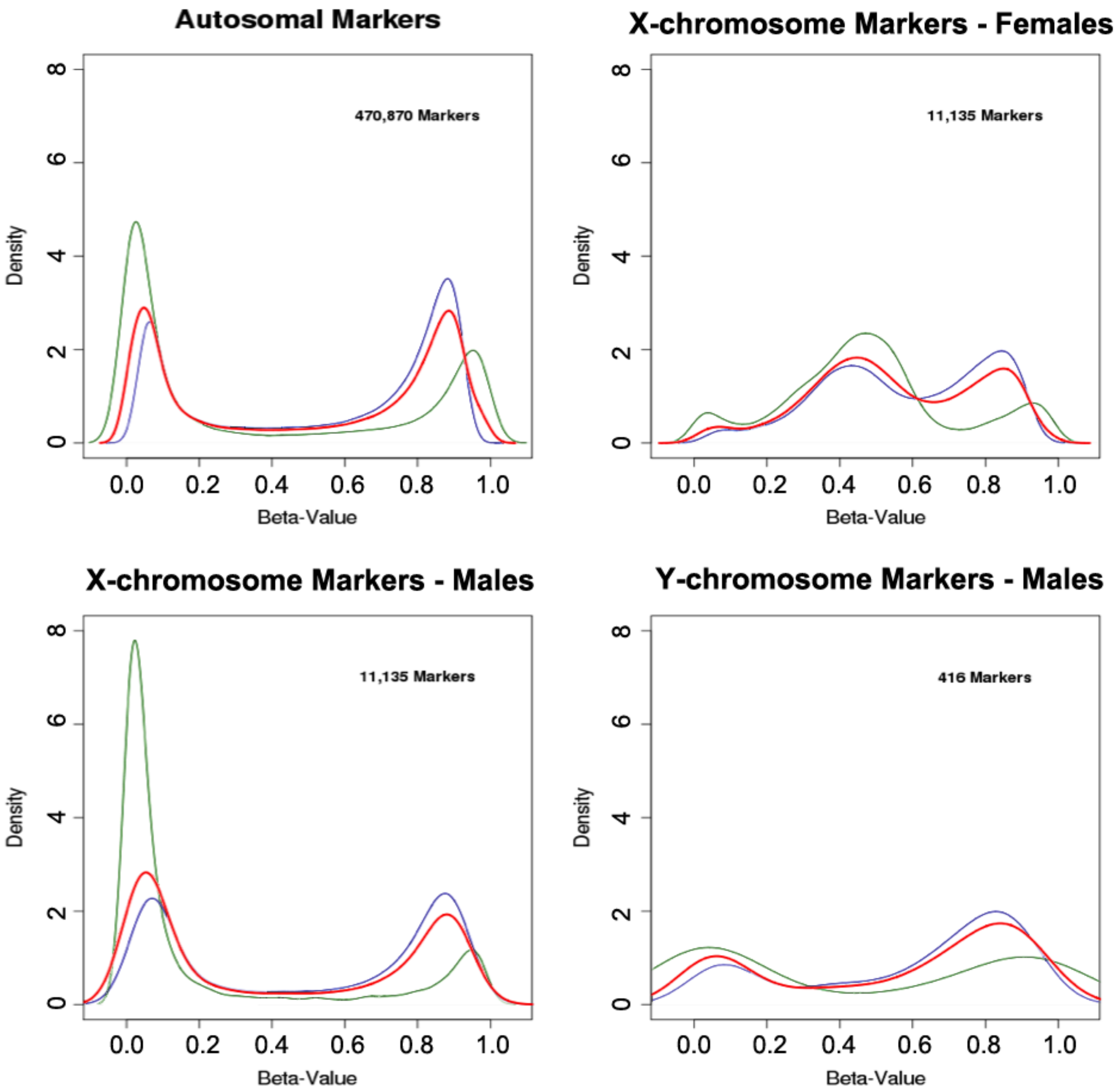

Supplementary Figure 2. Detection P-value.

SF 2A: distribution of marker intensities used for calculation of for detection P values

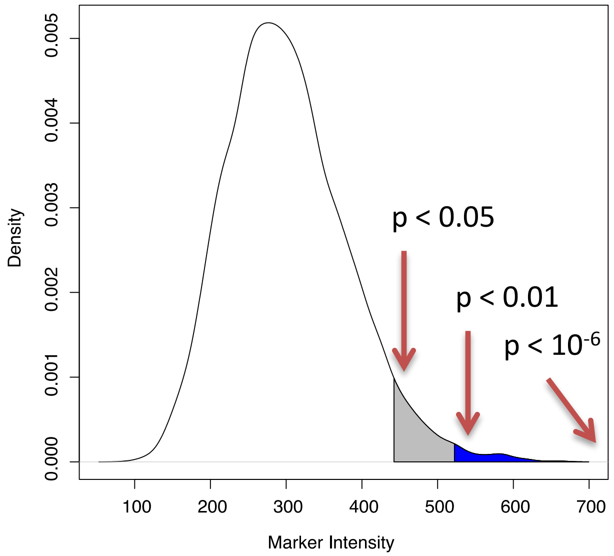

SF 2B: % of markers with missing data with different detection P –values. Minfi reports detection p-values smaller than 2.2 x 10^{-16} as 0 (smallest positive floating-point number x such that 1+x ≠ 1).

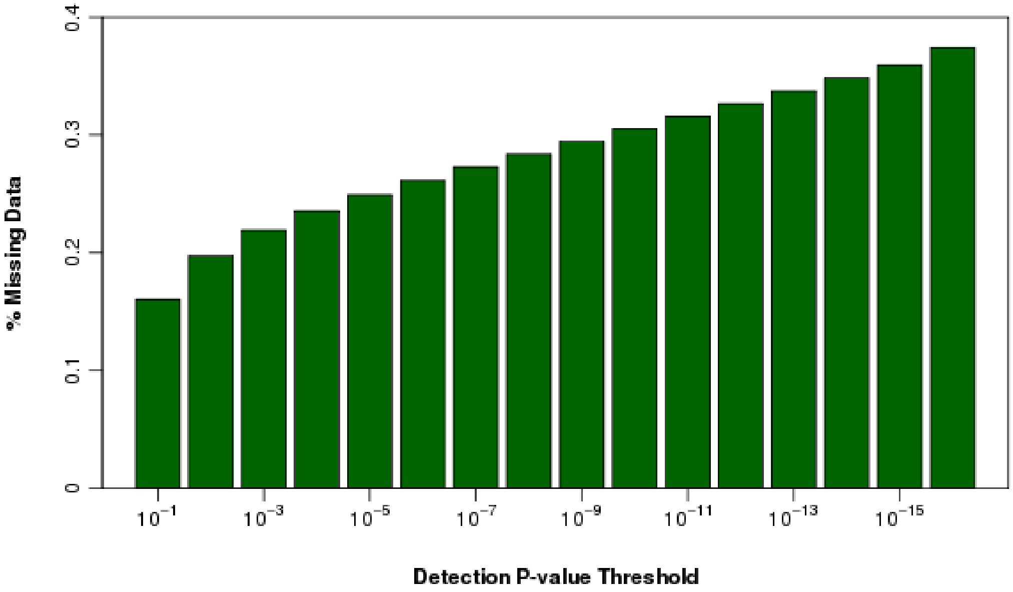

Supplementary Figure 3. Identifying the optimal detection P-value threshold. To choose the optimal detection P-value threshold, we compared the percentage of chromosome Y markers with non-zero call-rates in females (green line) to the percentage of successfully called chromosome Y markers in males (red line) at different detection P-value thresholds. Markers known to cross-hybridise were excluded. A more stringent detection P-value threshold reduces the percentage of markers with non-zero call-rates in females (False Positives) but has no material effect on maker call-rates in males (True Positives).

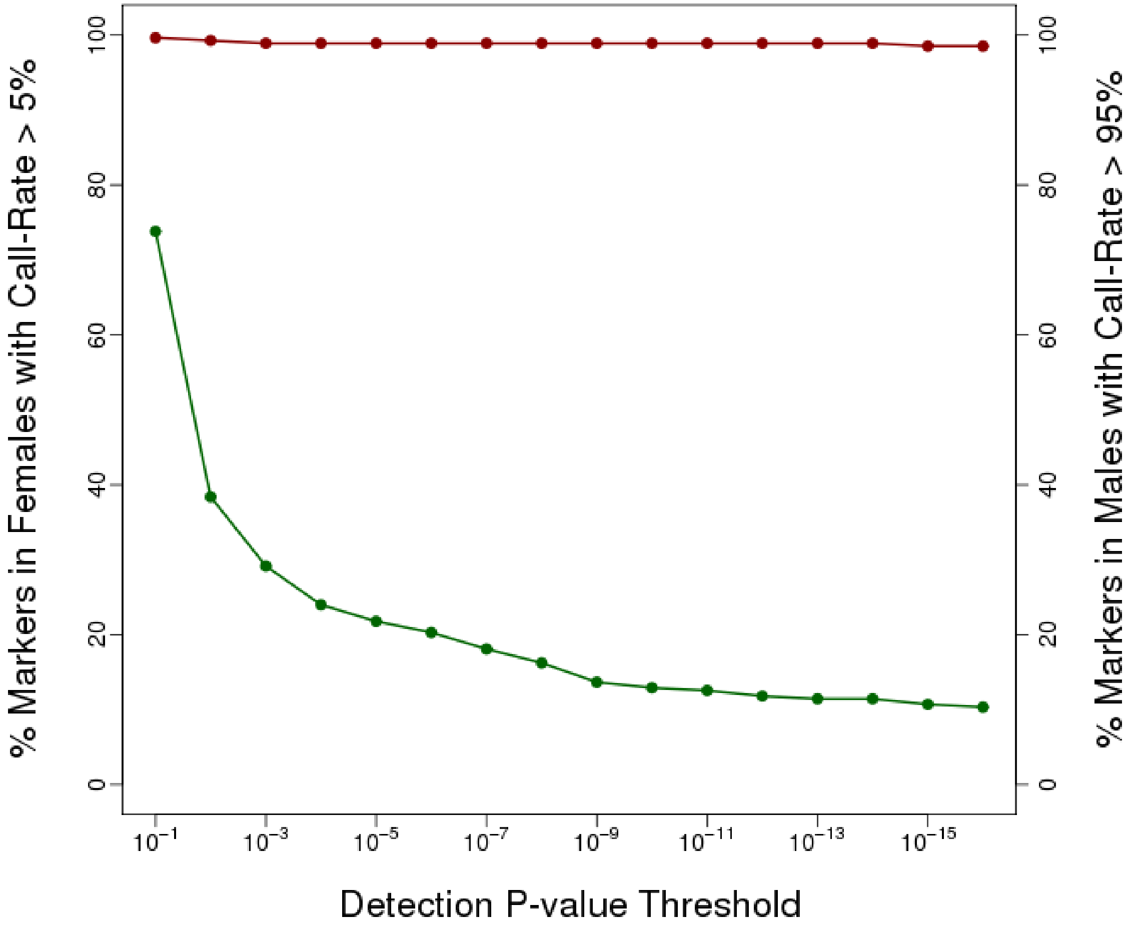

Supplementary Figure 4. Impact of detection P-value threshold on marker call rates (blue columns) and the proportion of values that are outliers (red dots). For a detection threshold of P<0.05 we observe a significant association between call rate and outlier rate ($P=1.40 \times 10^{-17}$; linear regression) which is not observed (P<0.142) at a detection threshold of $P<10^{-16}$.

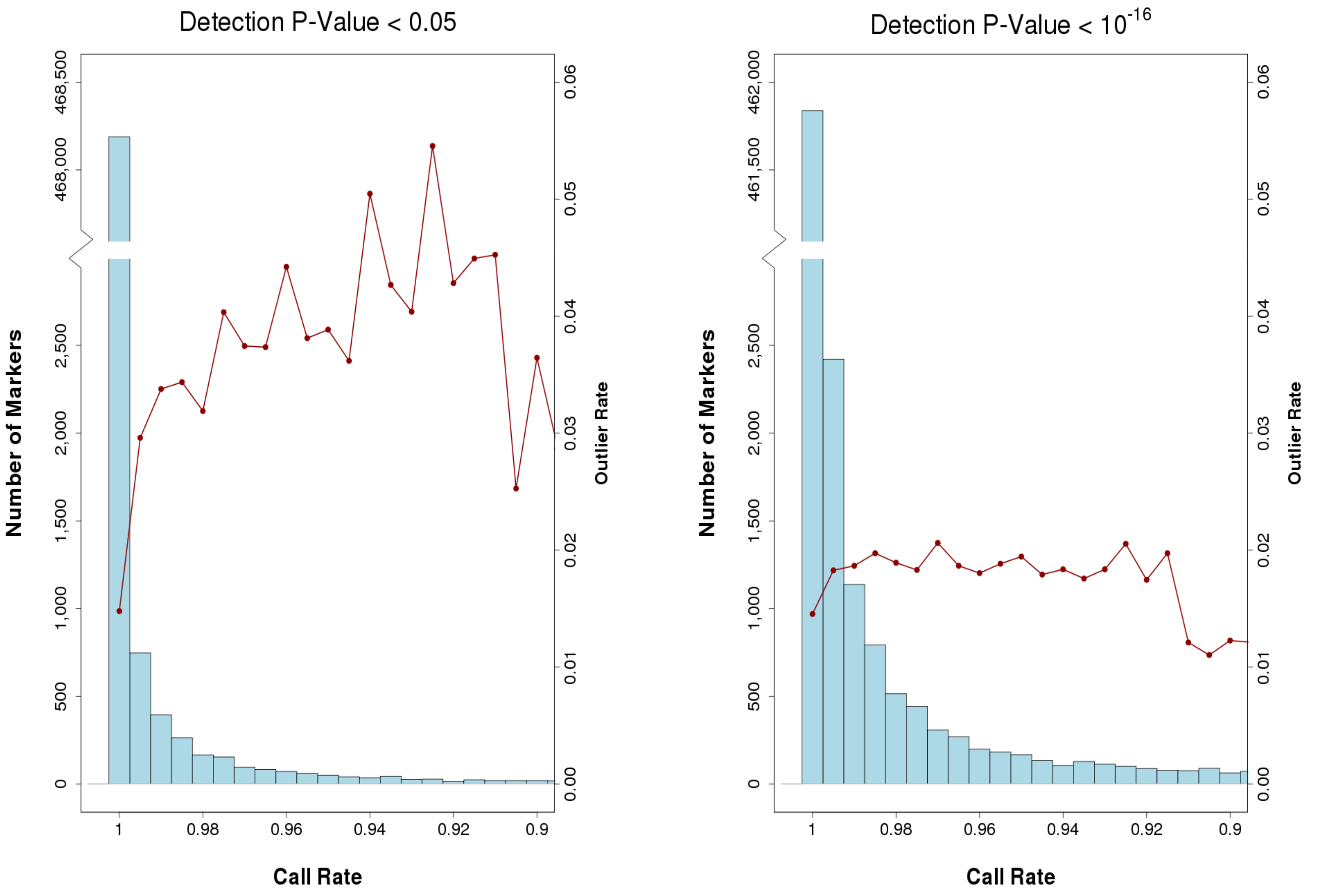

Supplementary Figure 5. Adoption of a stringent detection threshold ($P<10^{-16}$) significantly increases correlation between technical duplicates ($P < 2.91 \times 10^{-11}$, paired Wilcoxon test). Correlation coefficients are calculated between duplicate measurements of methylation in each sample considering 25,400 Methylation markers which show an altered call-rate after adoption of the more stringent threshold.

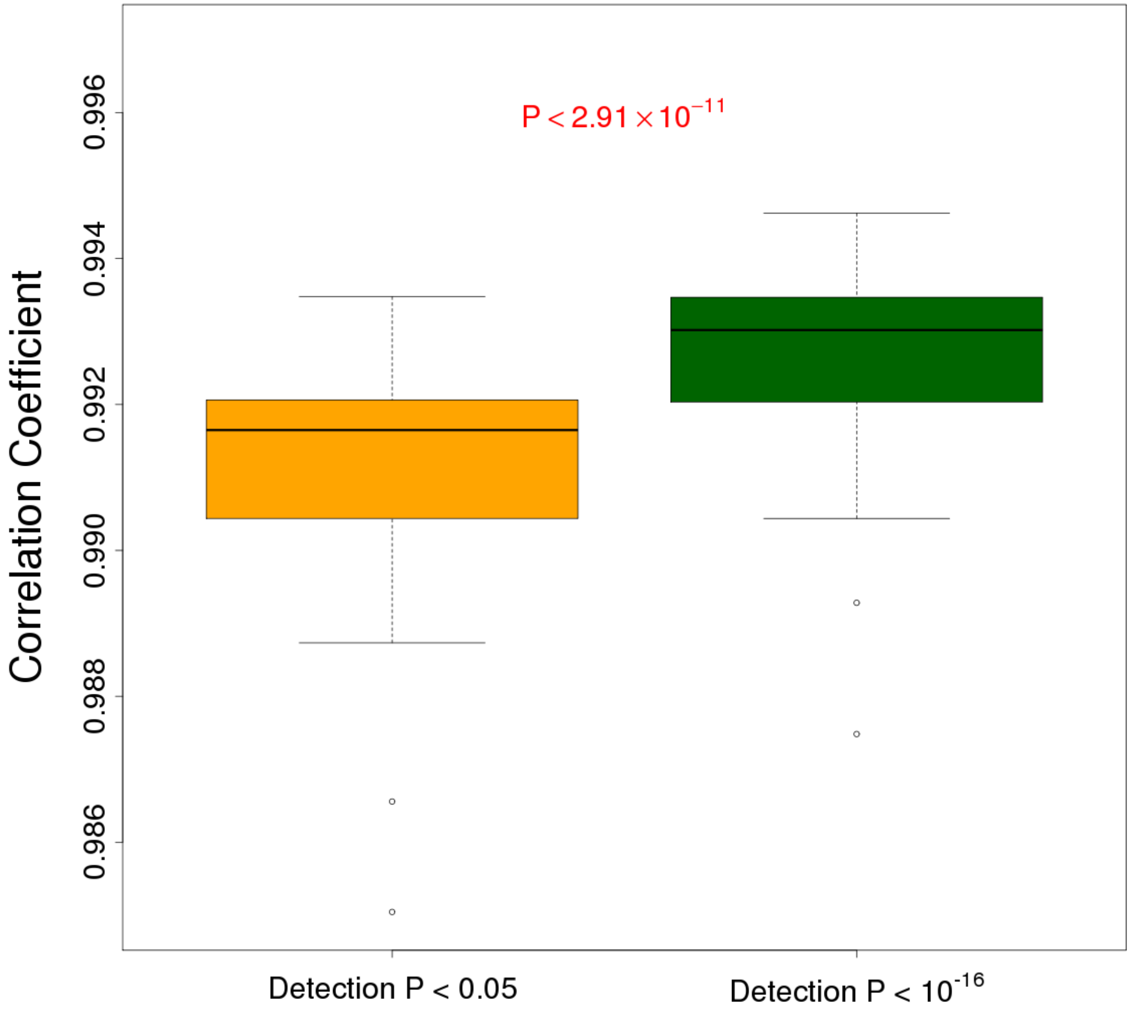

Supplementary Figure 6: Example of correlation between technical replicates on the sample and marker level. To identify the optimal normalisation method we compare methylation values (beta-values) using Pearson correlation coefficients. Correlation coefficients are calculated A) at the sample level to derive a correlation co-efficient between the paired measurements of the ~470,000 markers assayed in each of the 36 duplicate samples and B) at the marker level to derive a correlation coefficient between the 36 paired measurements for each of the ~470,000 markers assayed.

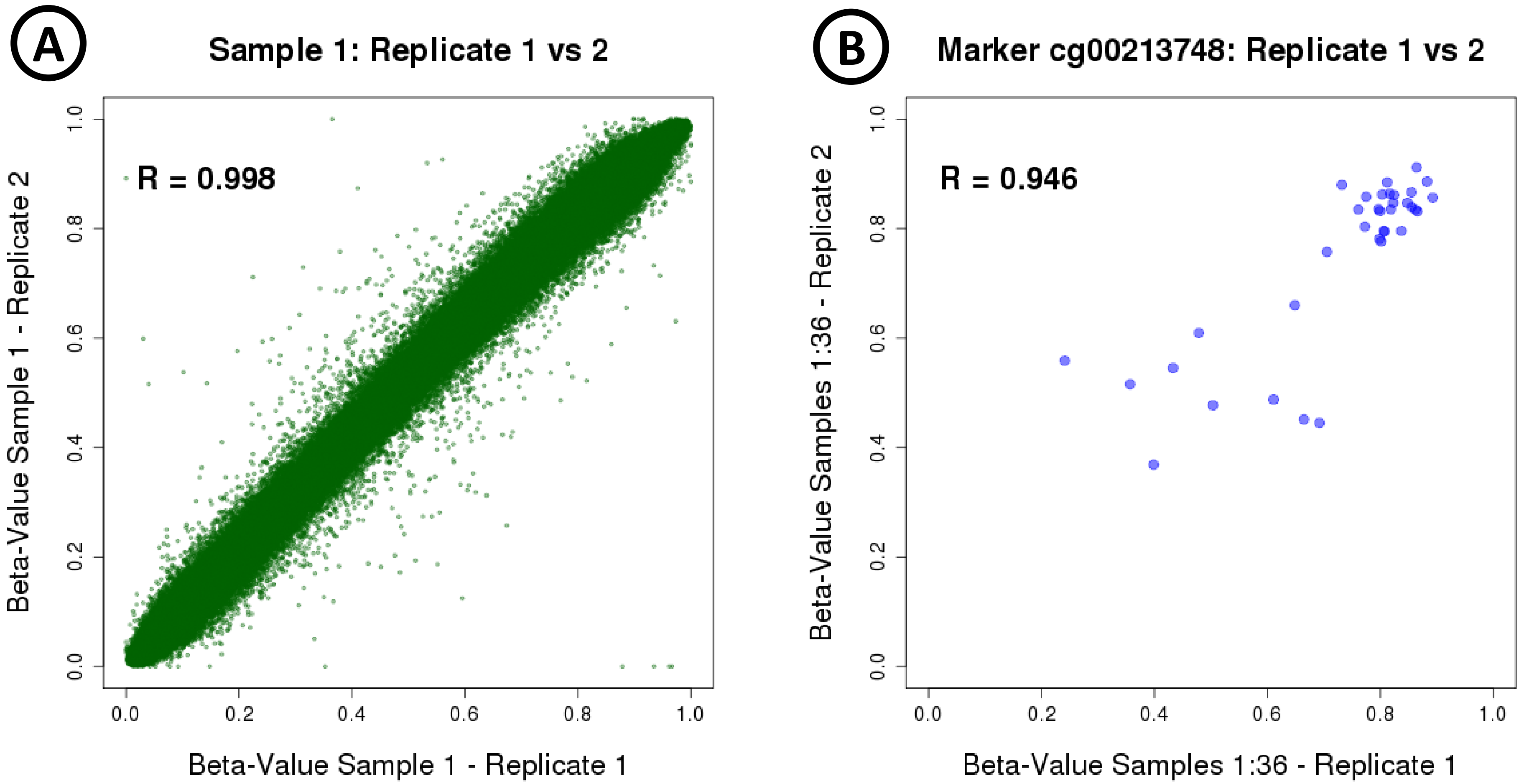

Supplementary Figure 7. Correlation between duplicate measurements of methylation in each sample, using different approaches to data-normalisation. Subset QN: Subset Quantile Normalization; Raw: No normalisation; BMIQ: Beta MIxture Quantile dilation; PBC: Peak-Based Correction; Illumina: Illumina Control Probe Normalisation; FN: Functional Normalisation; SWAN: Subset Within-Array Normalisation; QN-I2: Quantile Normalisation of Intensity-values (2 categories); QN-B: Quantile Normalisation of Beta-values; QN-I4: Quantile Normalisation of Intensity-values (4 categories); QN-I6: Quantile Normalisation of Intensity-values (6 categories). Methods are sorted based on the median correlation coefficient.

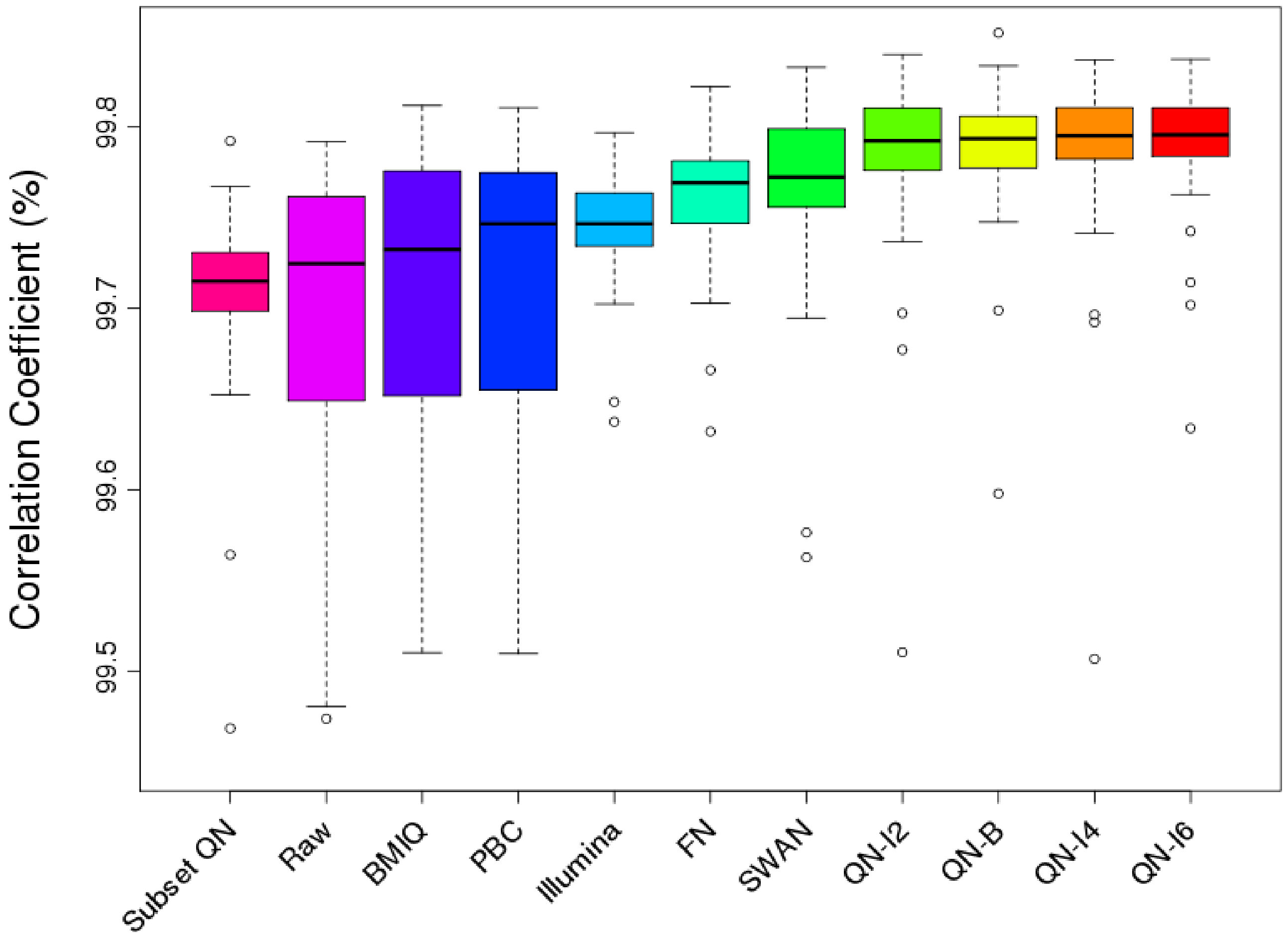

Supplementary Figure 8. Correlation between duplicate measurements at each methylation marker, using different approaches to data-normalisation. Raw: No normalisation; PBC: Peak-Based Correction; BMIQ: Beta MIxture Quantile dilation; FN: Functional Normalisation; SWAN: Subset Within-Array Normalisation; QN-B: Quantile Normalisation of Beta-values; Subset QN: Subset Quantile Normalization; QN-I2: Quantile Normalisation of Intensity-values (2 categories); Illumina: Illumina Control Probe Normalisation; QN-I4: Quantile Normalisation of Intensity-values (4 categories); QN-I6: Quantile Normalisation of Intensity-values (6 categories). Methods in the figure legend are sorted based on the median correlation coefficient.

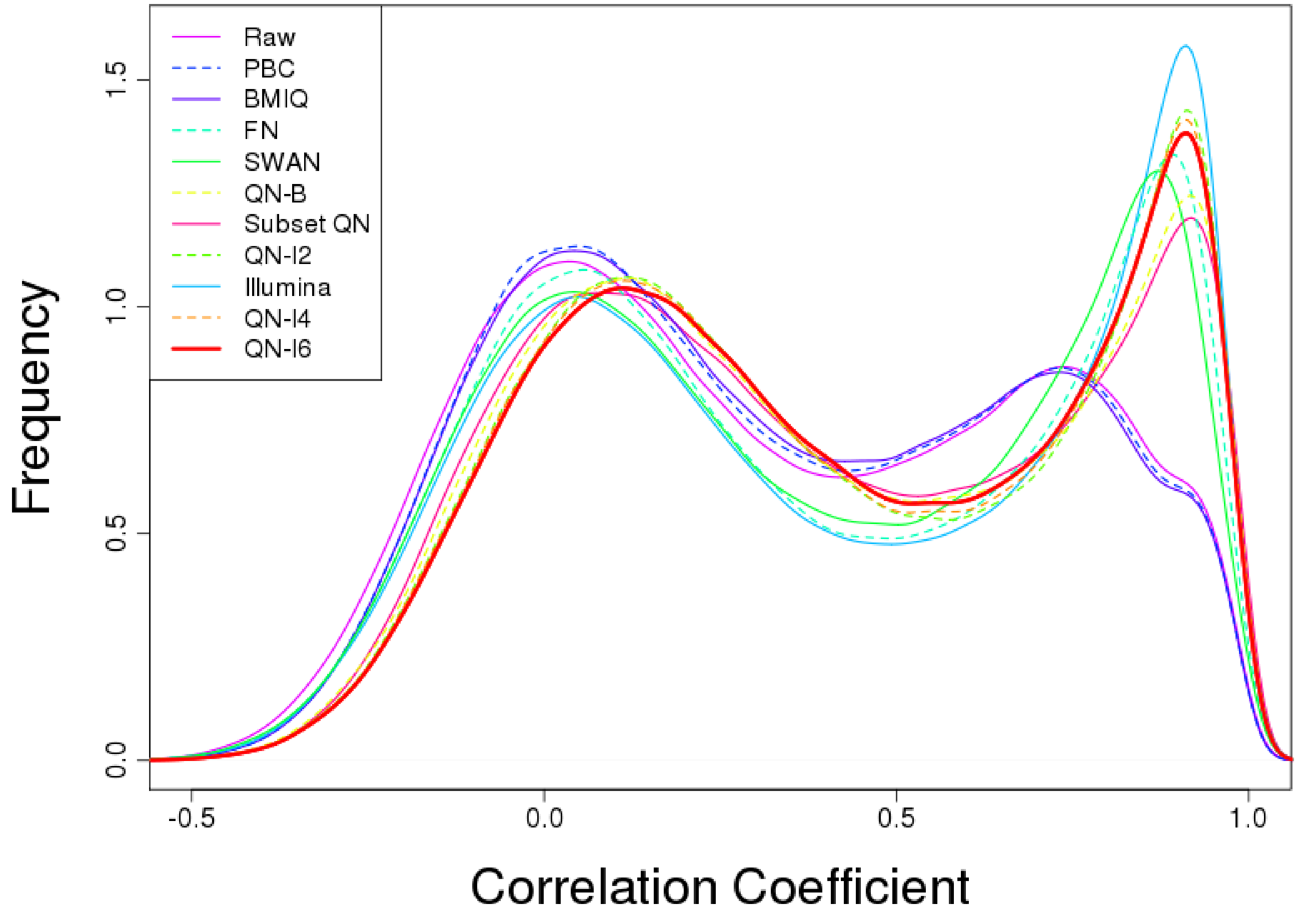

Supplementary Figure 9: Simulation analysis comparing different approaches to data normalisation. For each normalisation method we increased (“spiked”) beta-values of 100 randomly selected markers and determined the proportion of spiked markers that were ranked amongst the top 100. SWAN: Subset Within-Array Normalisation; Illumina: Illumina Control Probe Normalisation; Raw: No normalisation; BMIQ: Beta MIxture Quantile dilation; PBC: Peak-Based Correction; FN: Functional Normalisation; Subset QN: Subset Quantile Normalization; QN-B: Quantile Normalisation of Beta-values; QN-I2: Quantile Normalisation of Intensity-values (2 categories); QN-I4: Quantile Normalisation of Intensity-values (4 categories); QN-I6: Quantile Normalisation of Intensity-values (6 categories). Methods in the figure legend are sorted based on the average percentage of top 100 spike-markers.

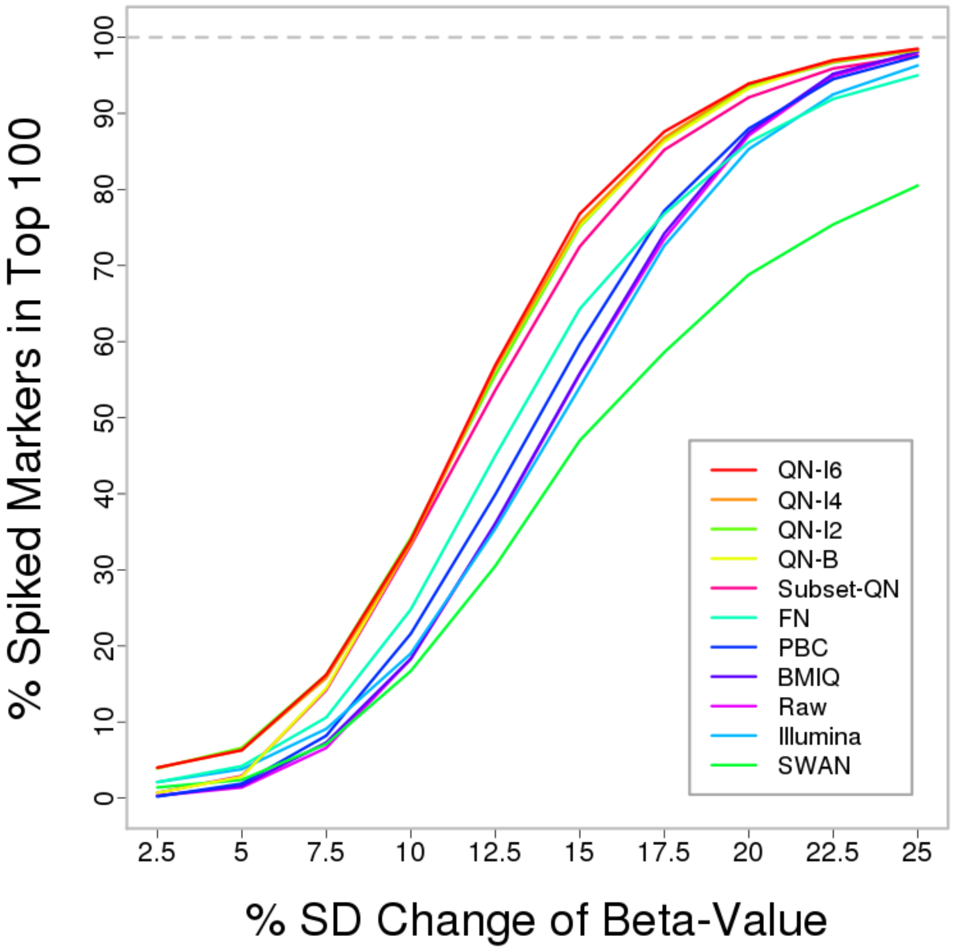

Supplementary Figure 10: Correcting for statistical inflation due to technical biases using *champ.runCombat*. Quantile-Quantile (QQ) plot for the comparison of 36 samples measured in duplicate reveals considerable statistical inflation after quantile normalisation (λ_{QN}=2.11; blue points). Batch correction using *champ.runCombat* (λ_{CB}=0.97; orange points) partially decrease technical biases, but substantial statistical inflation remains.

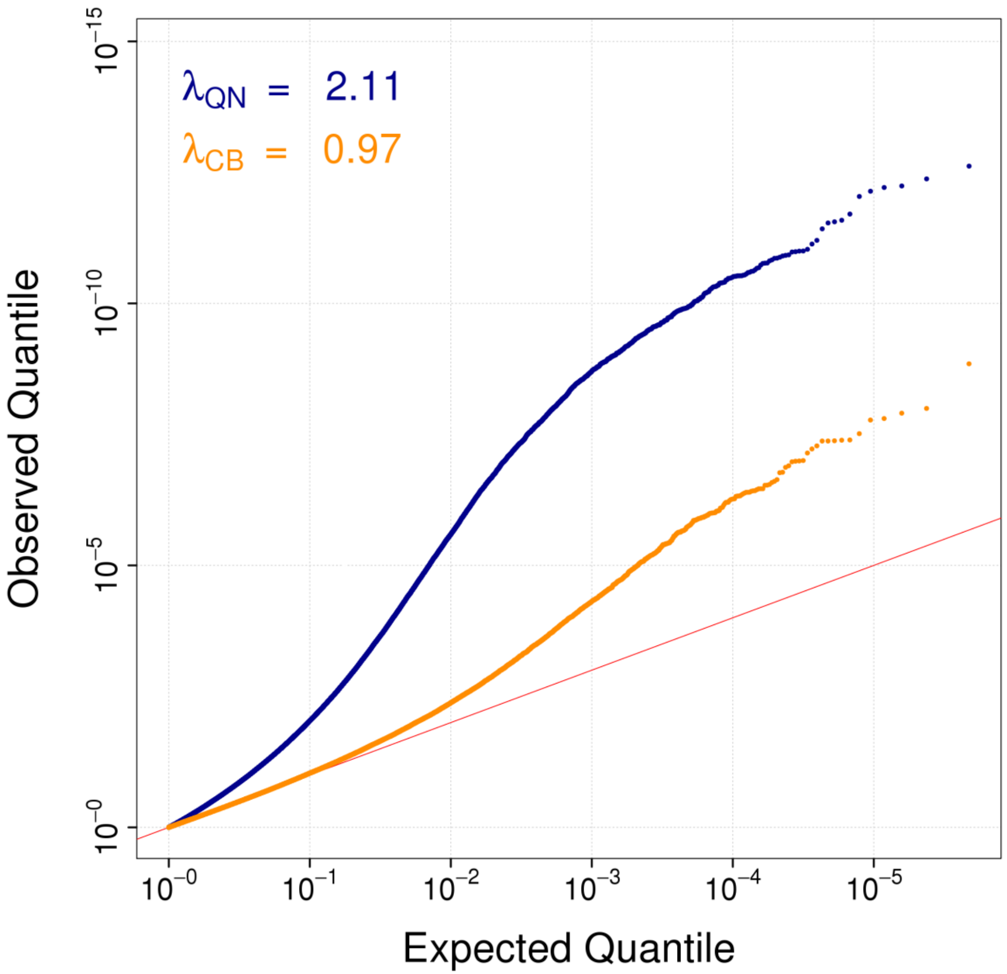

Supplementary Figure 11. Correlation between control probes. Heatmap of Pearson Correlation Coefficients between 220 control probes on the Infinium 450K methylation array.

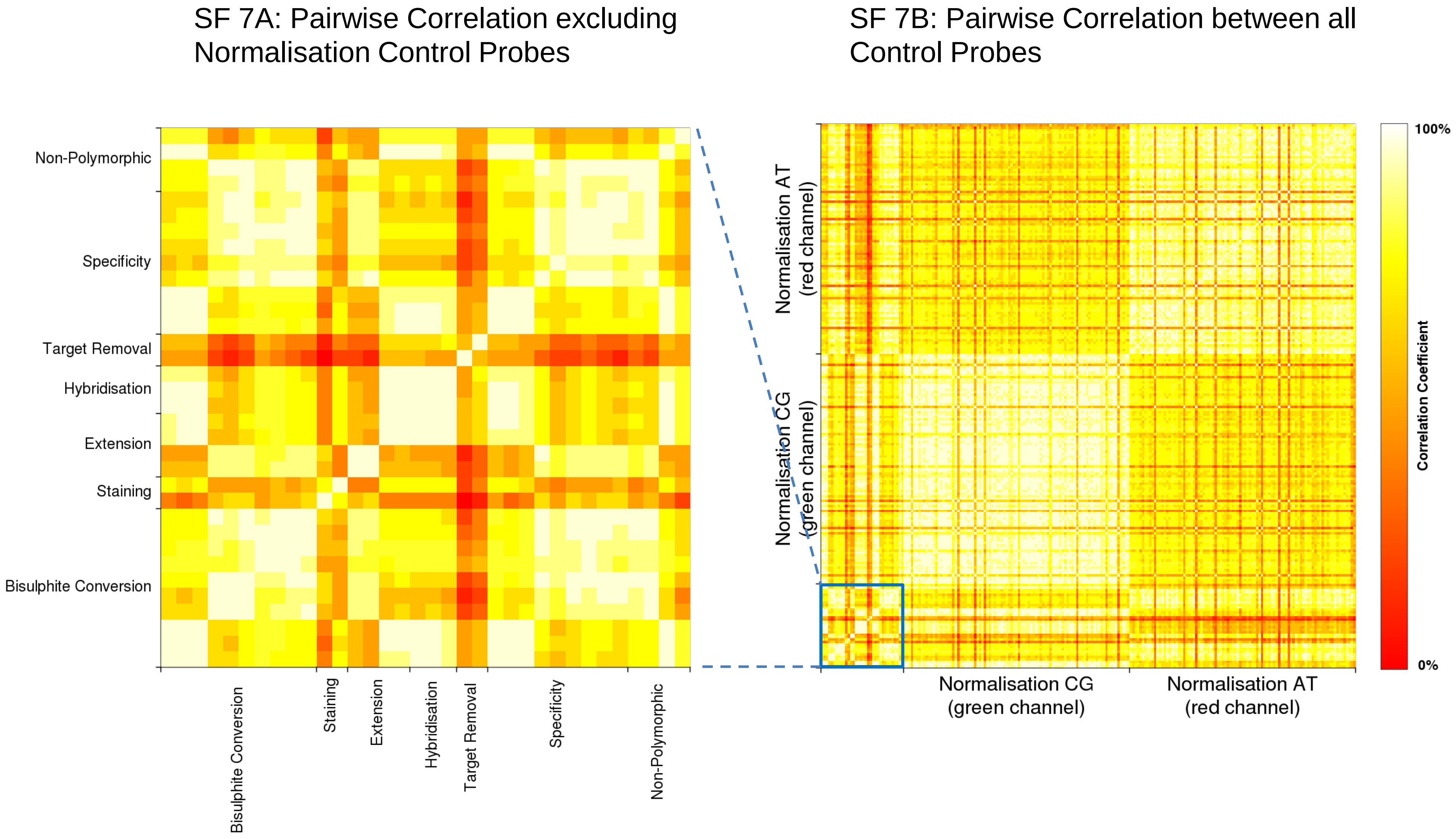

Supplementary Figure 12. PCA of control probe intensities reveals strong correlations between Principal Components and technical factors in the population study.

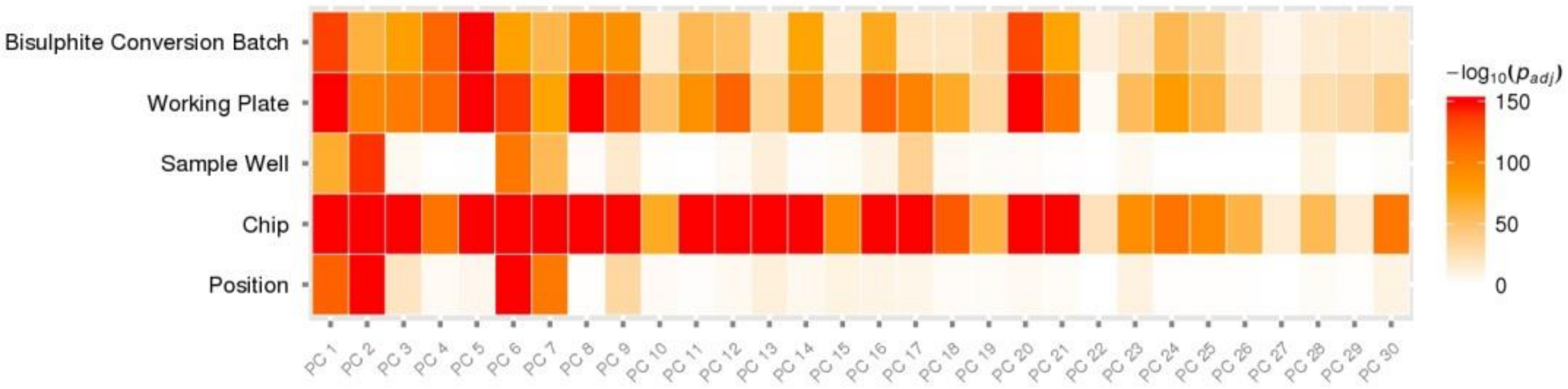

Supplementary Figure 13. Correction for control probes removes batch effects in the technical replication dataset.

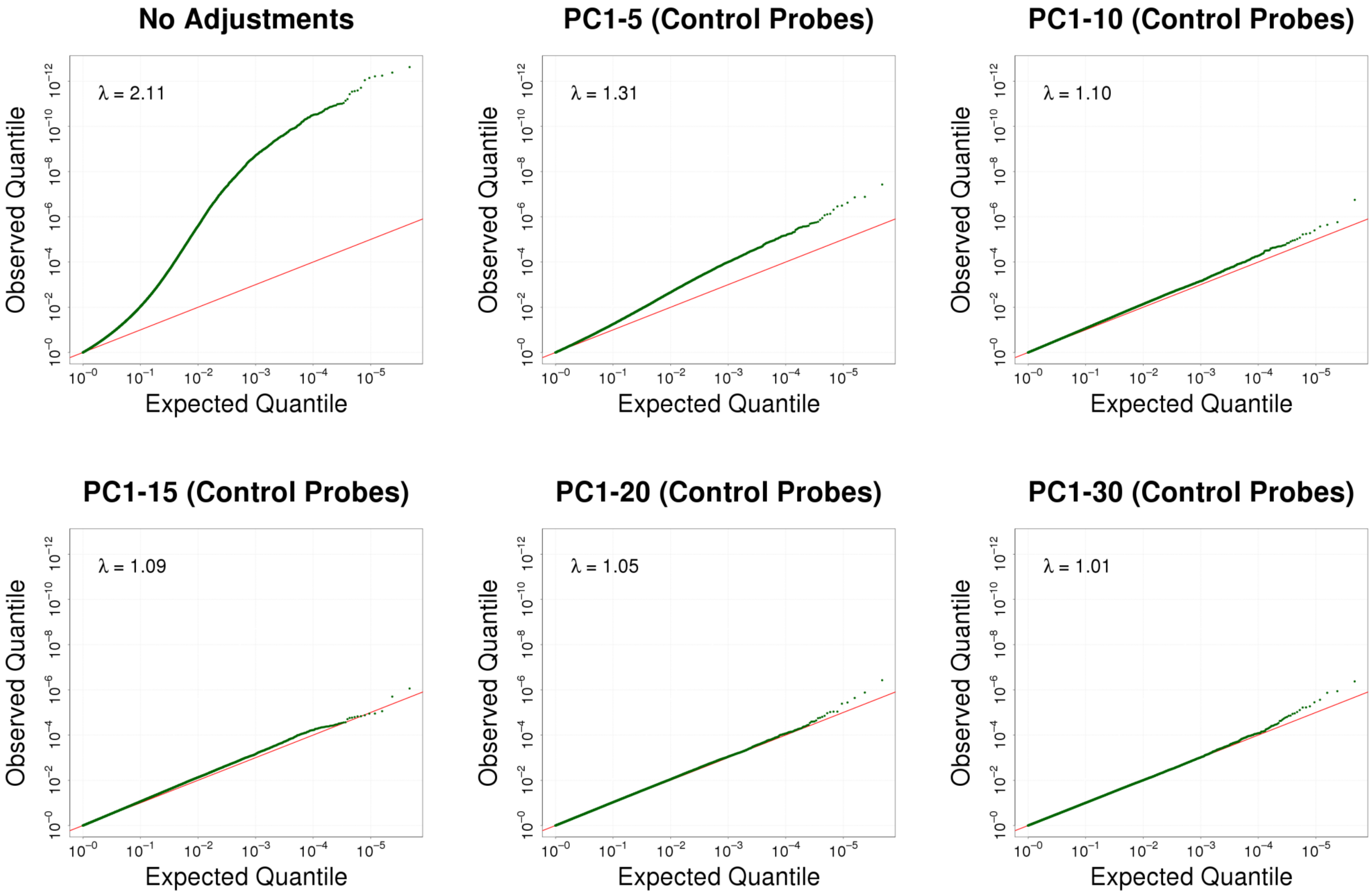

Supplementary Figure 14. PCA before and after adjustment for control probes showing that correction for control probes effectively removes technical biases in the population study.

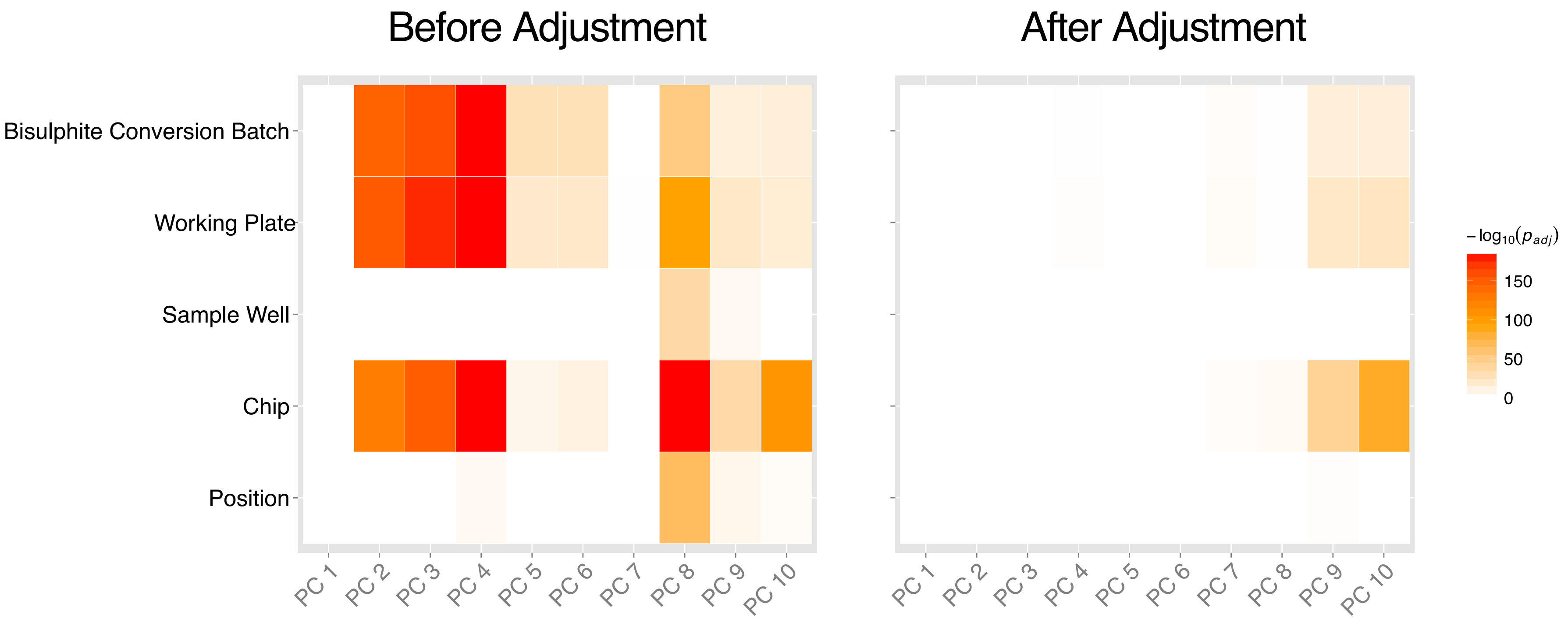

Supplementary Figure 15. Distribution of pairwise correlation between 1000 randomly selected markers.

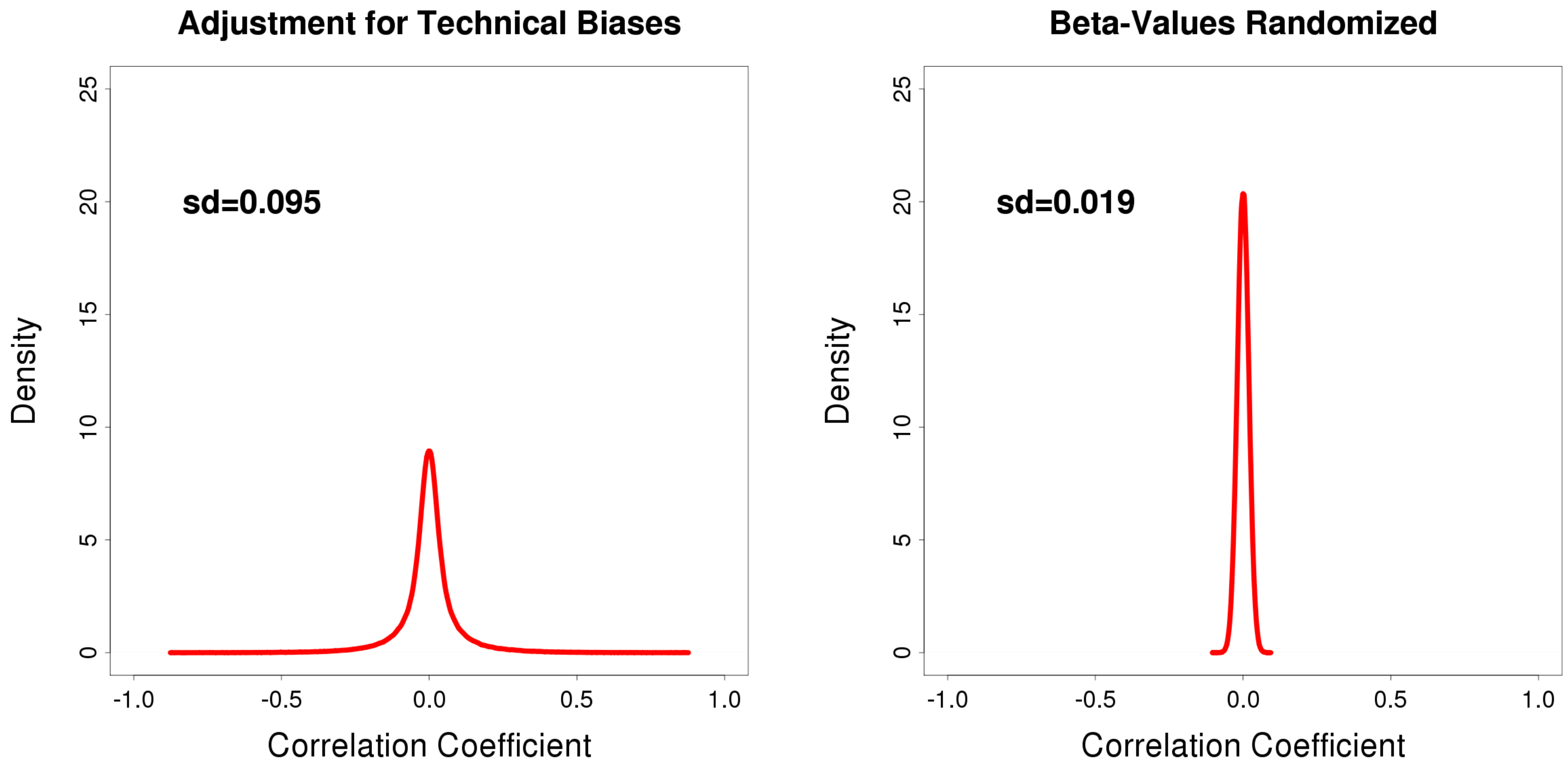

Supplementary Figure 16. Test statistic distribution under the null hypothesis by permutation testing. A) Prediction interval after QN and control probe adjustment. Correlation between markers results in an overall deflation of test statistics and a broad prediction interval. B) Randomly reassigning beta-values for each marker re-establishes independence resulting in a narrow prediction interval around the expected.

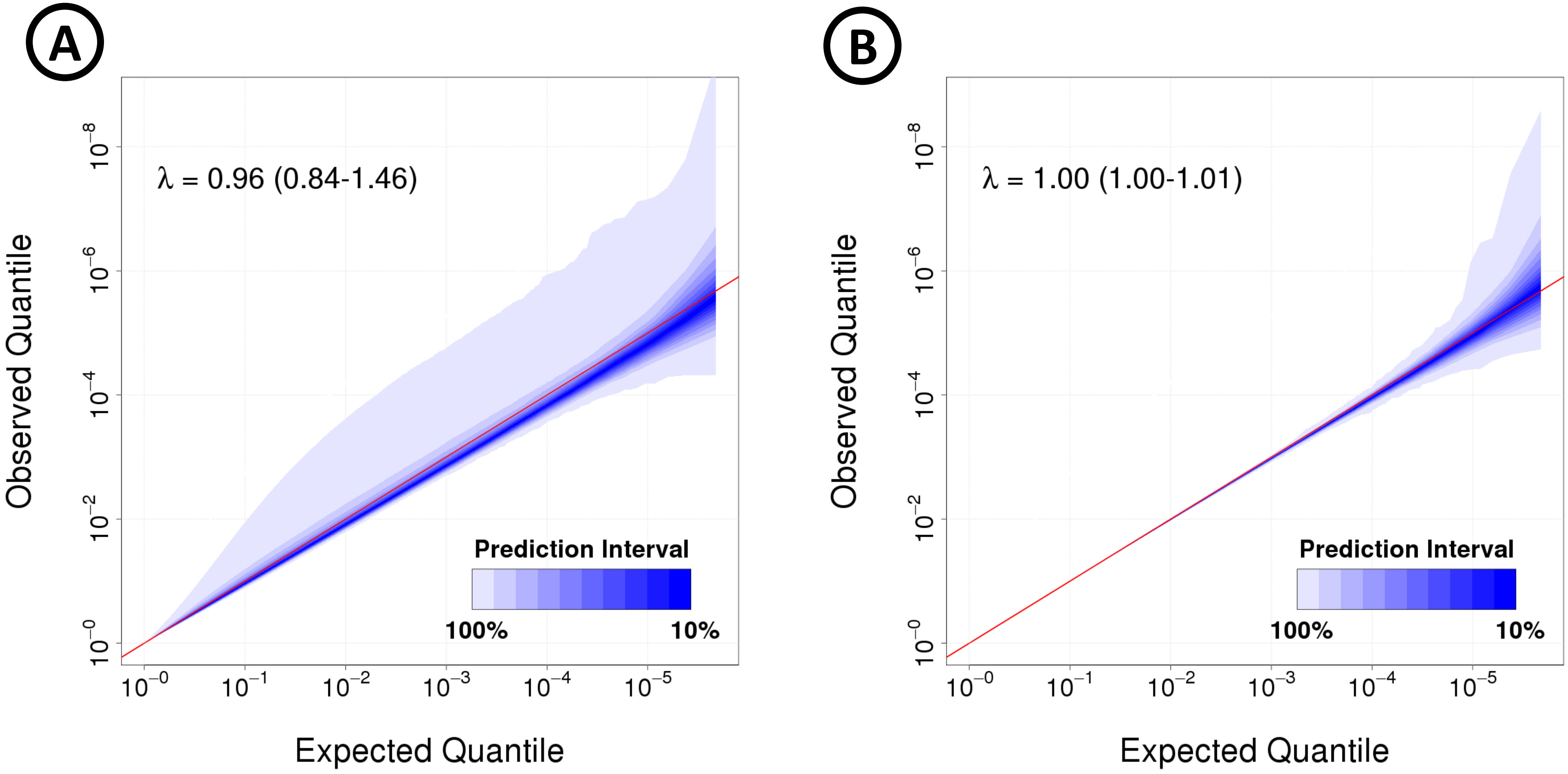

Supplementary Figure 17. Estimated white-blood cell subsets accurately reproduce measured white-blood cell subsets. Measured granulocytes: sum of the basophil, eosinophil and neutrophils; imputed lymphocytes: sum of CD4+, CD8+, NK cell and B cell.

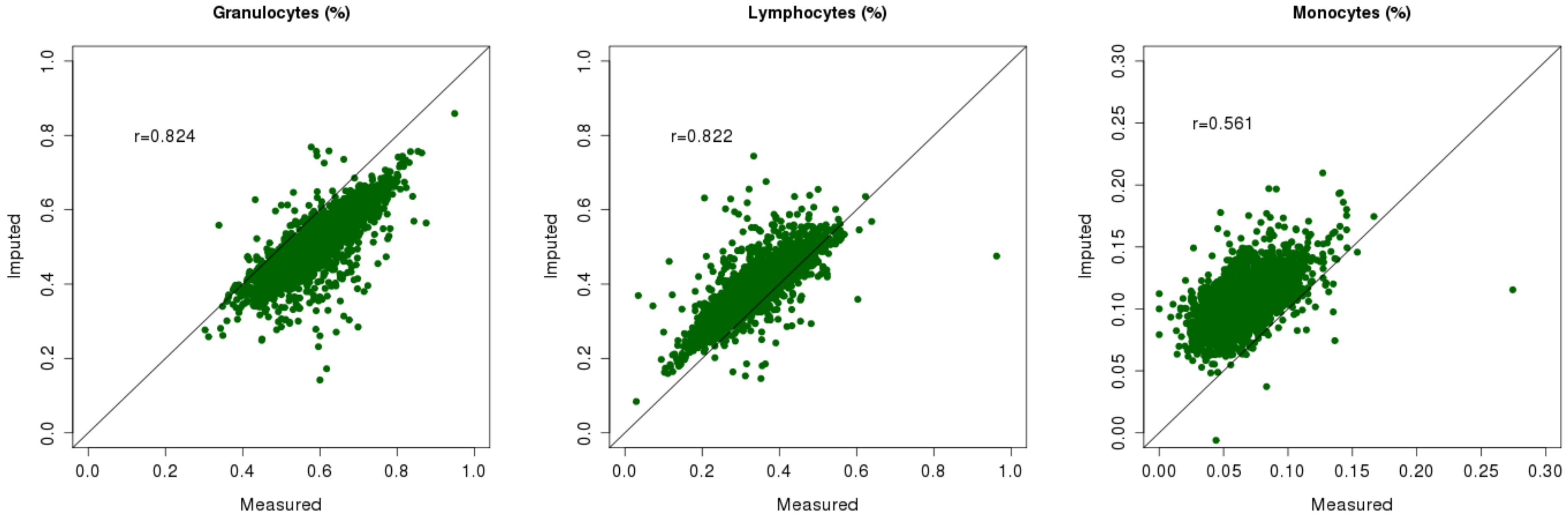

Supplementary Figure 18. Test statistic distribution under the null hypothesis by permutation testing, using different levels of adjustment.

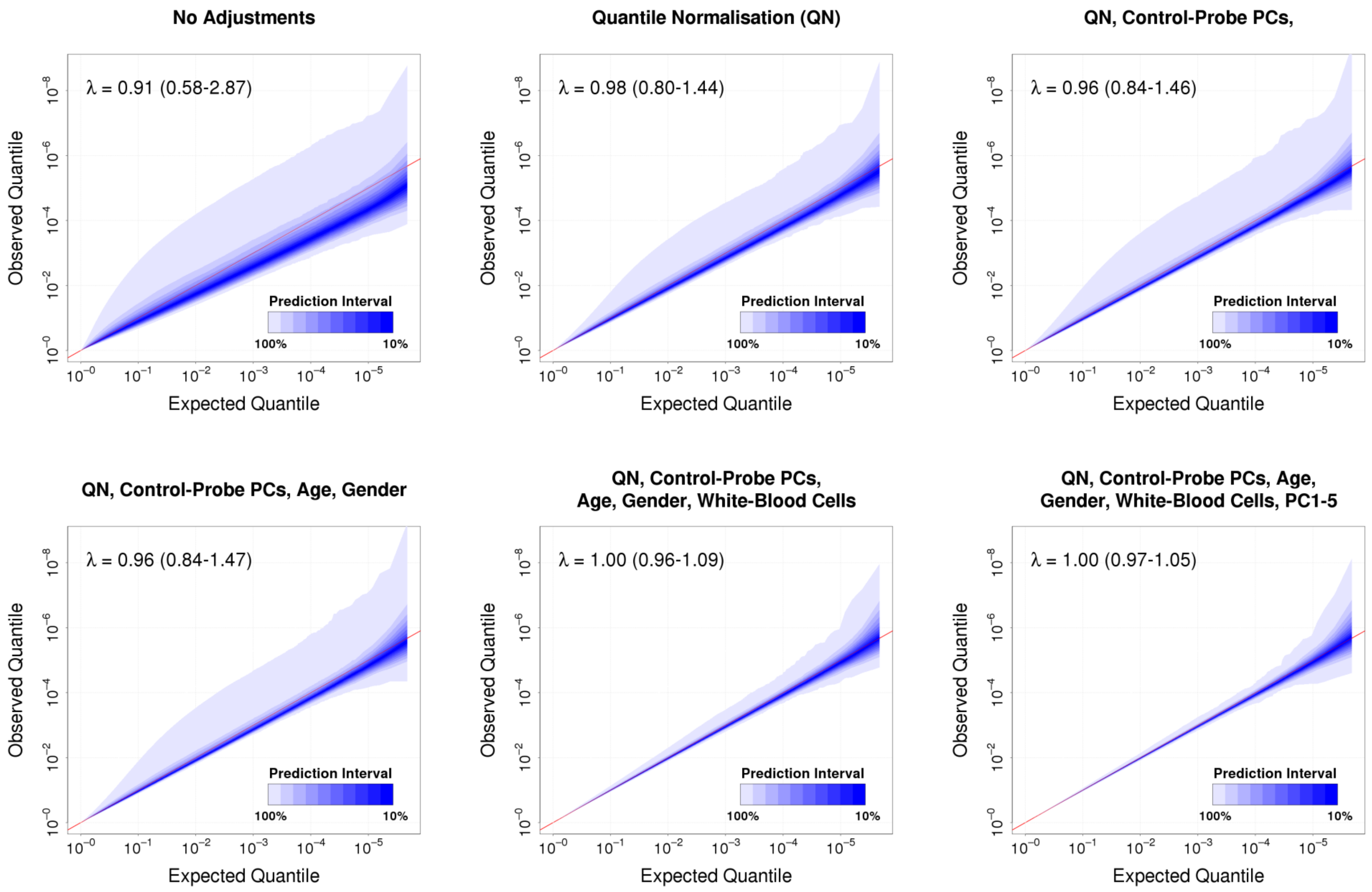

Supplementary Figure 19. Correlation between 1,000 randomly chosen markers under different levels of adjustment. We perform a linear regression predicting the beta-value as a function of the various linear predictors and calculate Pearson Correlation Coefficients between marker pairs based on the residuals.

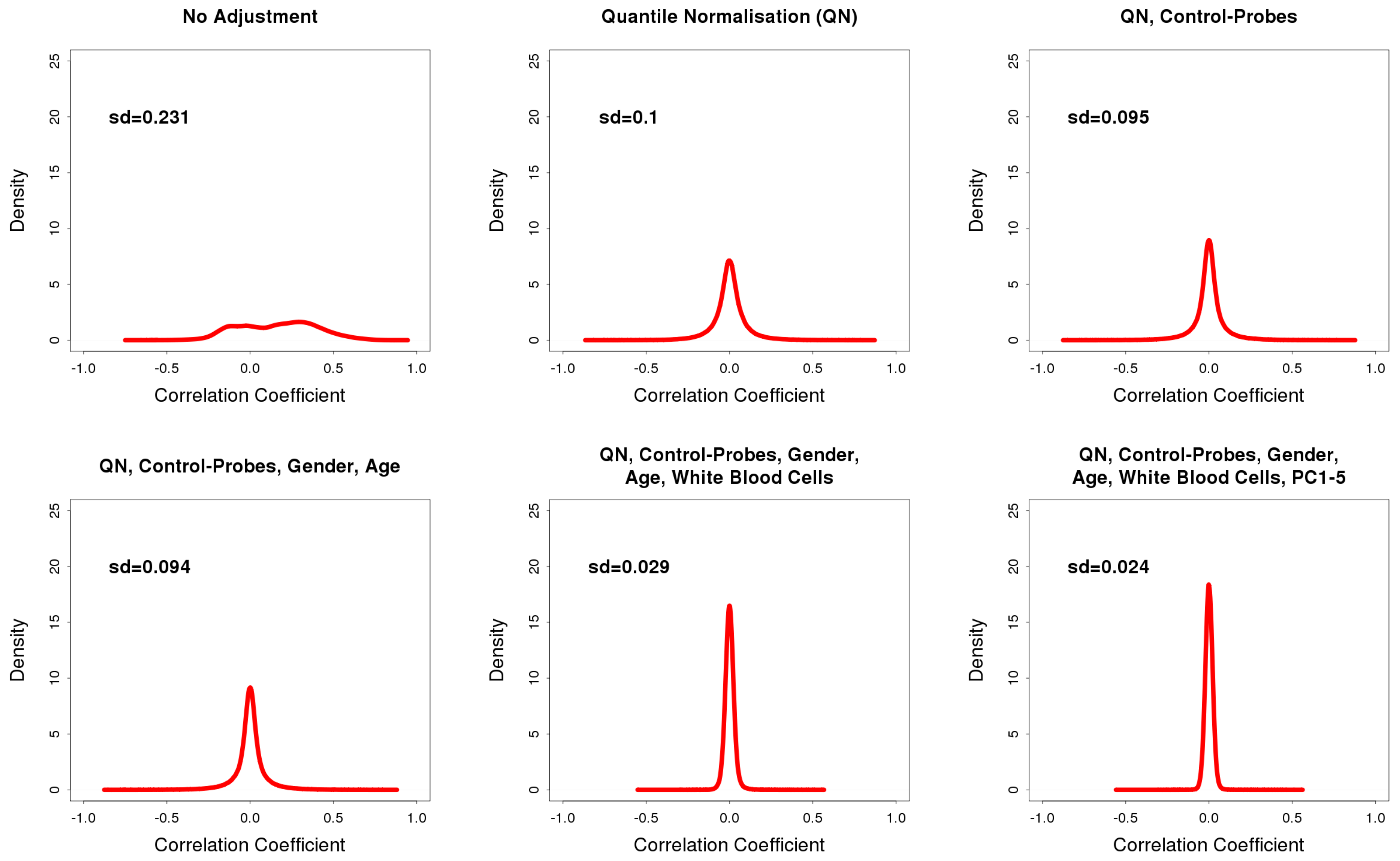

Supplementary Figure 20. Variance explained by Principal Components (PCs) 1-20 after Quantile Normalisation and adjustment for control probe PCs, age, gender and white-blood cells.

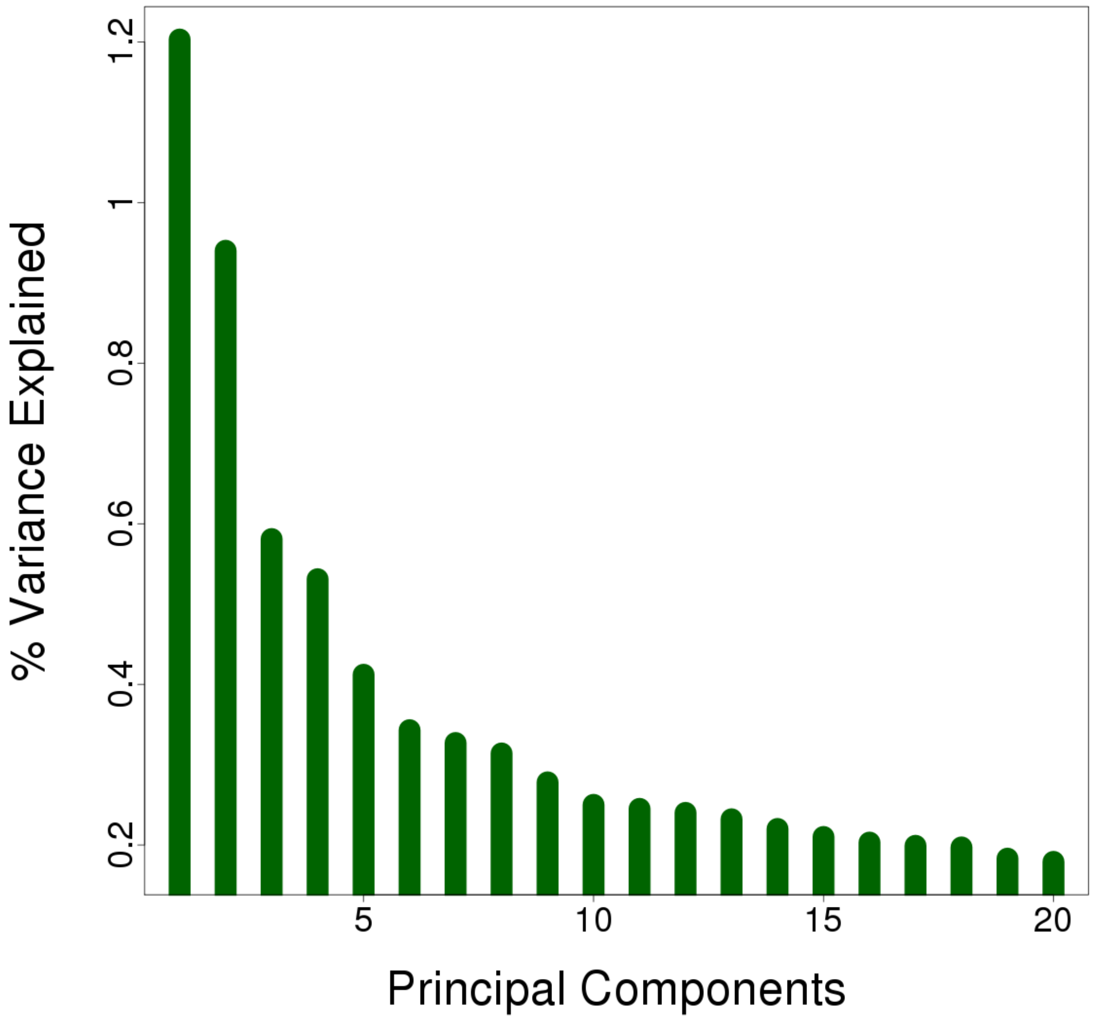

Supplementary Figure 21. Adjustment for for technical and biological factors reduces global, but retains local correlation. Blue shading represents the density of marker-pairs considering all autosomal markers, whereby correlation coefficients were calculated based on methylation-residuals after Quantile Normalisation (QN) and adjustment for control probe PCs, age, gender, white-blood cells and PC1-5. Lines are derived using a 300bp sliding window of the 5% most variable marker and are based on i. raw beta values (black), ii. residuals after QN and adjustment for control probe PCs (red) and iii. residuals after QN and adjustment for control probe PCs, age, gender, white-blood cells and PC1-5 (green). Adjustments proposed by the CPACOR pipeline preferentially reduce correlation between markers with a high distance ($P < 2.2 \times 10^{-308}$ for red vs black line and green vs black line; see methods for details)

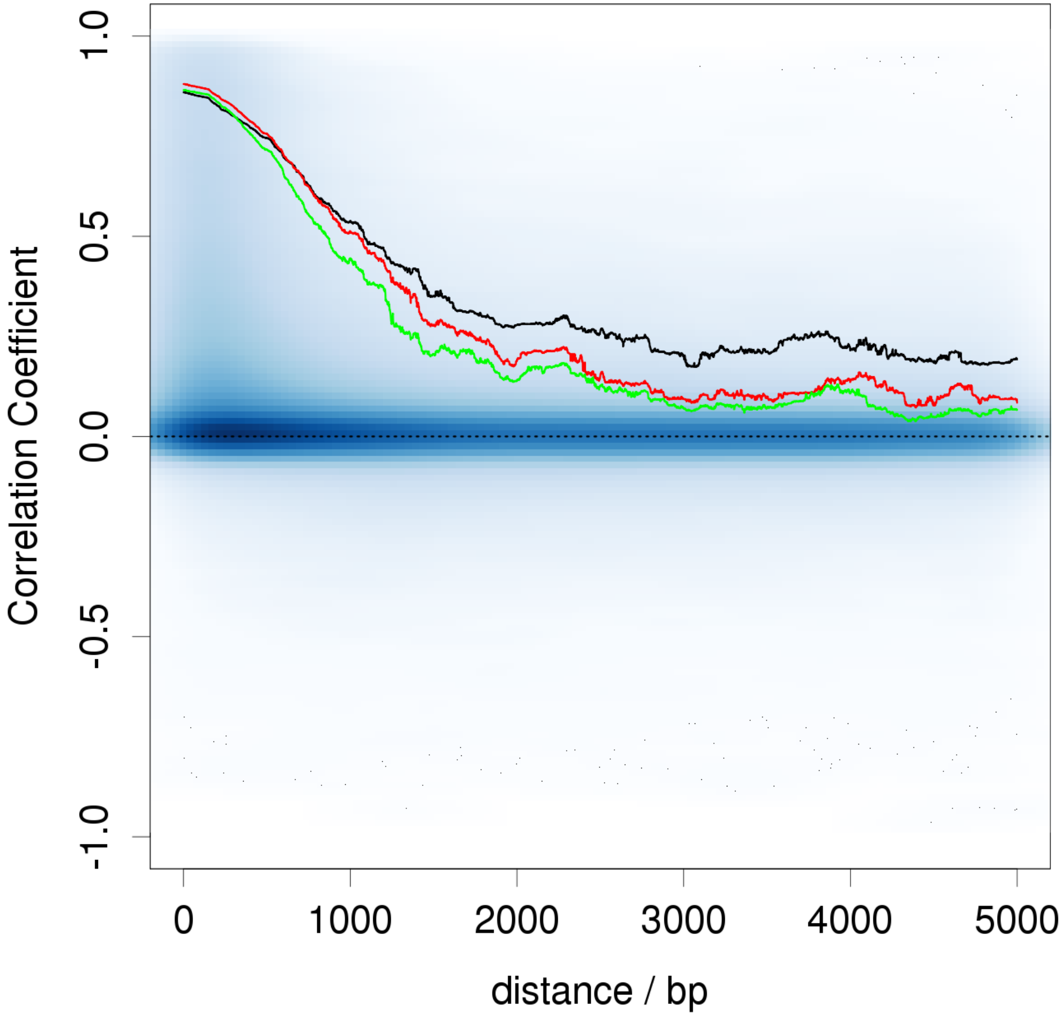

Supplementary Figure 22. Simulation analysis comparing CPACOR with existing analysis pipelines. We increased ("spiked") beta-values of 100 randomly selected markers and determined the proportion of the spiked markers that were ranked amongst the top 100 for each pipeline. A) Comparison of CPACOR with existing analysis pipelines for the complete dataset of 2,664 samples; only CPACOR and minfi completed the analysis successfully. B) Comparison of CPACOR with existing analysis pipelines for a reduced dataset of 500 samples.

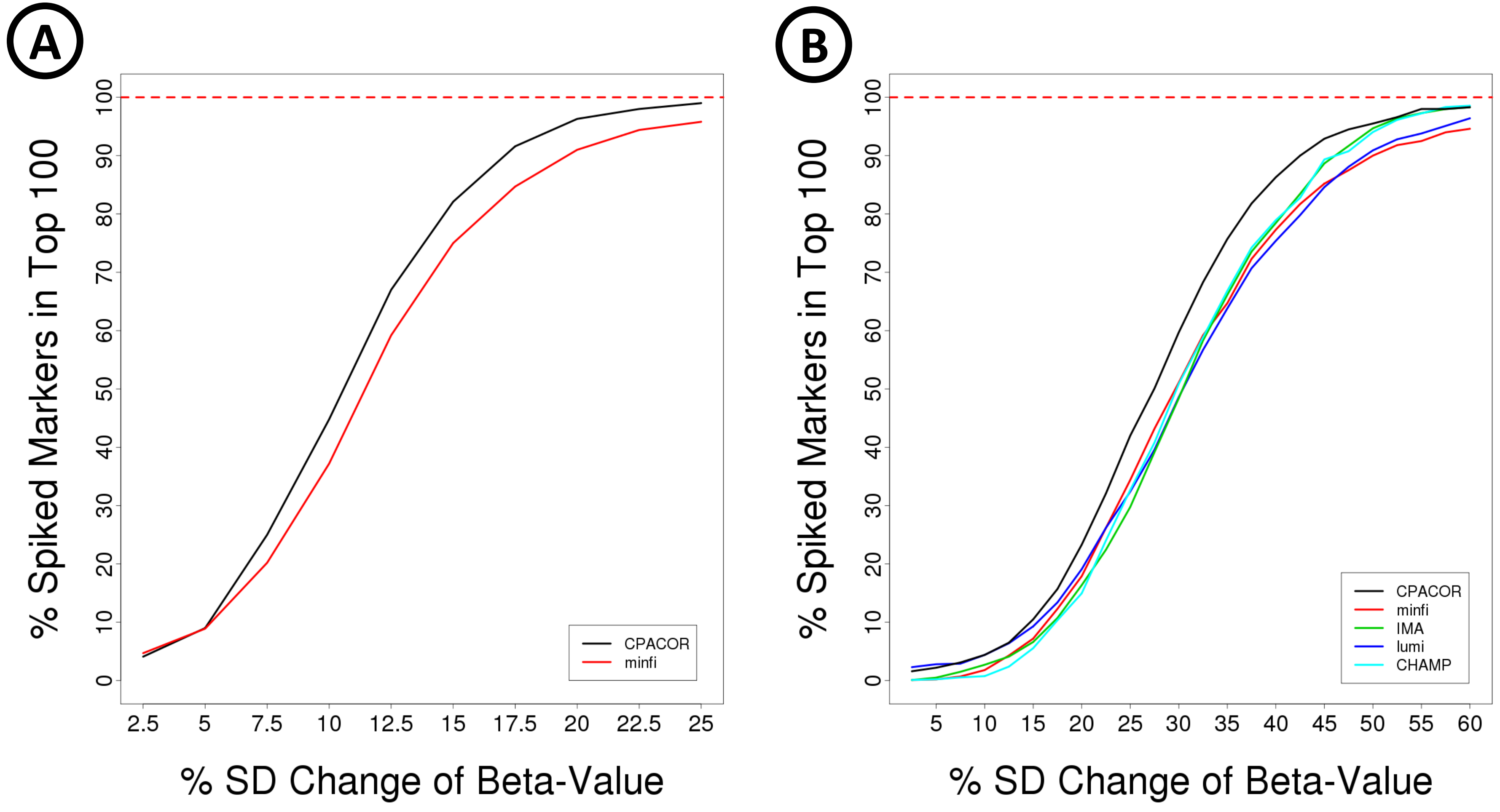

Supplementary Figure 23: Simulation analysis shows that EWASher-adjustment results in a reduction of performance. We increased (“spiked”) beta-values of 100 randomly selected markers at 10%, 30% and 50% SD of the respective beta-value and determined the proportion of the spiked markers that were ranked amongst the top 100 for successive stages of the EWASher approach. The proportion of spiked markers achieving top 100 ranks is reduced considerably after adjustment for the methylation similarity matrix.

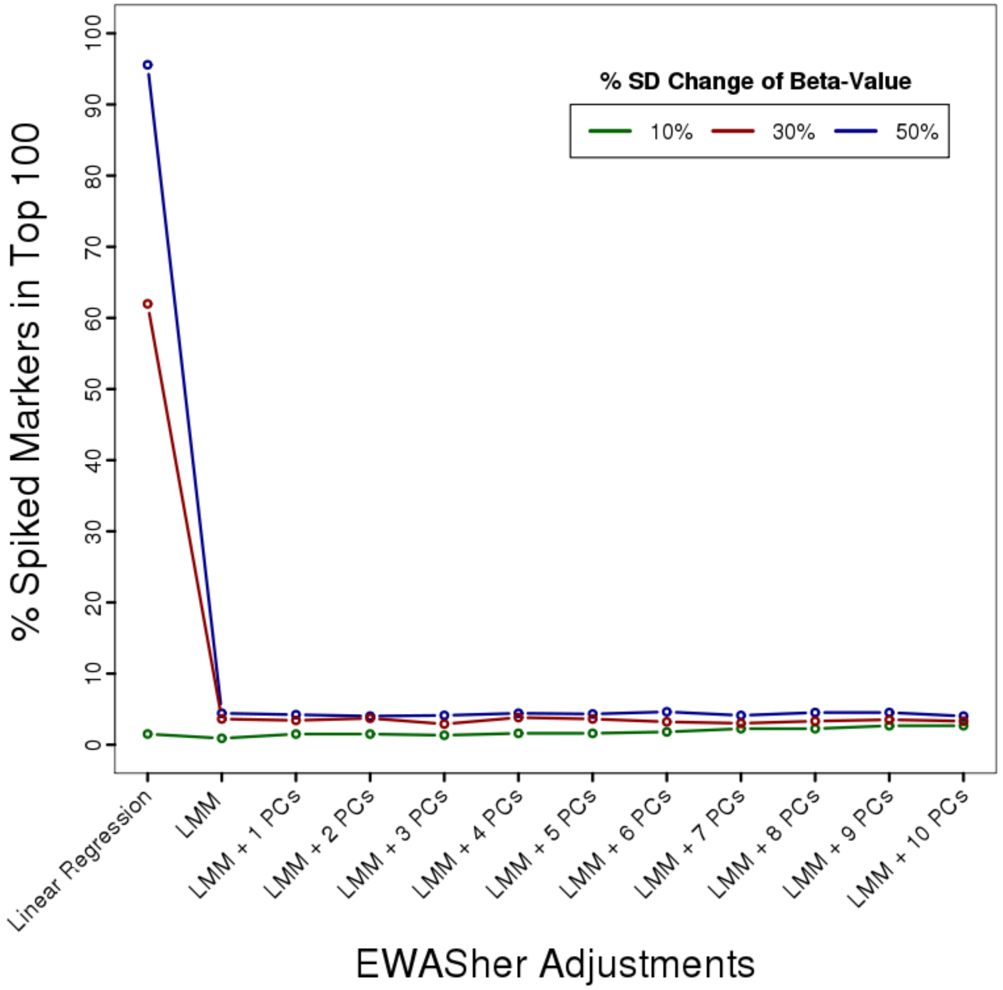

Supplementary Figure 24: Comparison of adjusted and unadjusted beta-coefficients derived using RefFreeEWAS. We increased ("spiked") beta-values of 100 randomly selected markers at 50% SD of the respective beta-value and performed EWAS analysis using the RefFreeEWAS R package. A) Adjusted and unadjusted beta-coefficients are compared for non-spiked (grey dots) and spiked (red dots) markers. B) Ranks of spiked markers are compared for RefFreeEWAS P-values (50 bootstraps), RefFreeEWAS beta-coefficents (adjusted) and CPACOR.

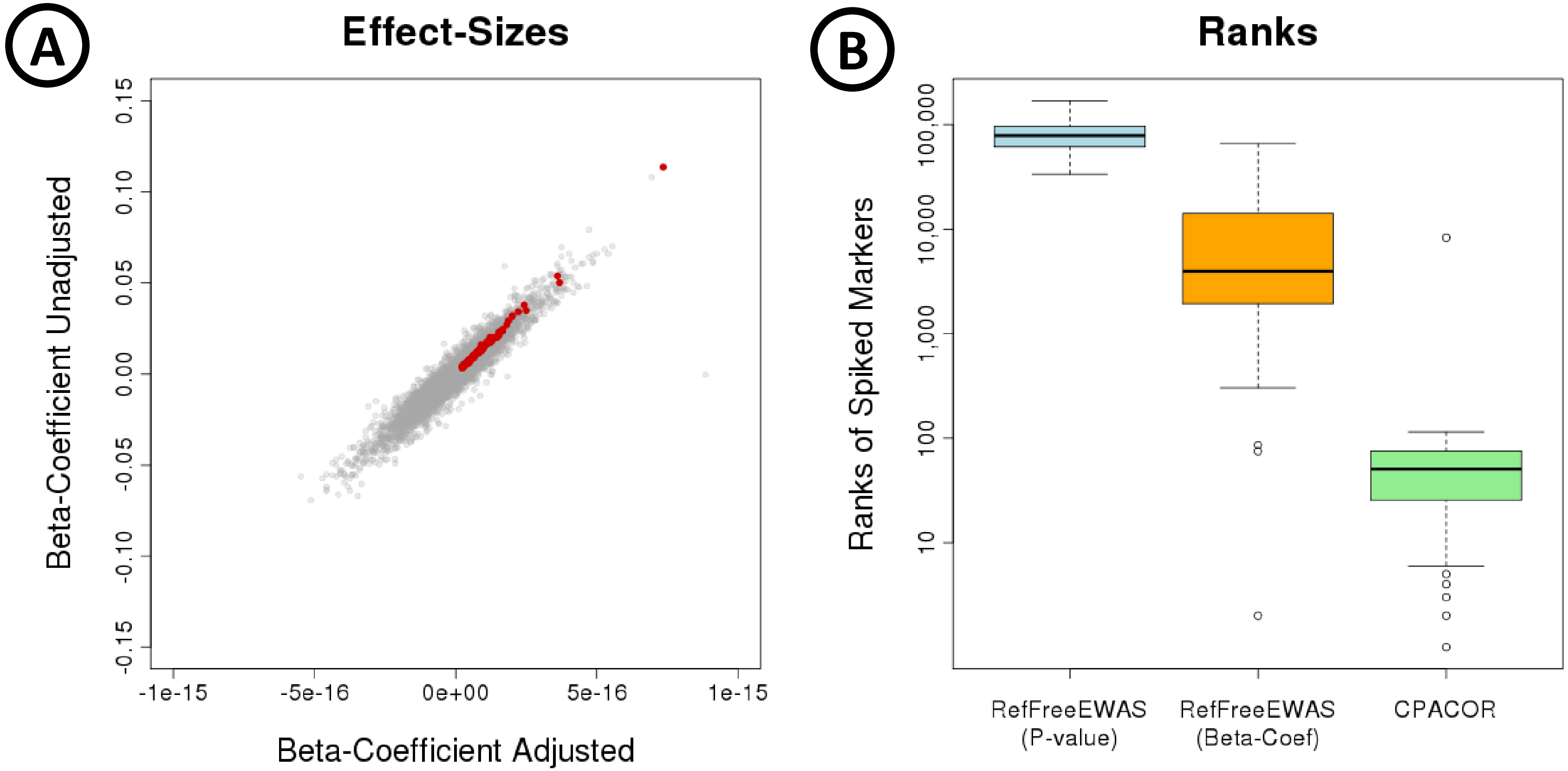

Supplementary Figure 25A. Correlation between 1,000 randomly chosen markers from three marker categories. We perform a linear regression predicting the beta-value (QN) as a function of control probe PCs, age, gender, white-blood cells and PC1-5 and calculate Pearson correlation coefficients between marker pairs based on the residuals.

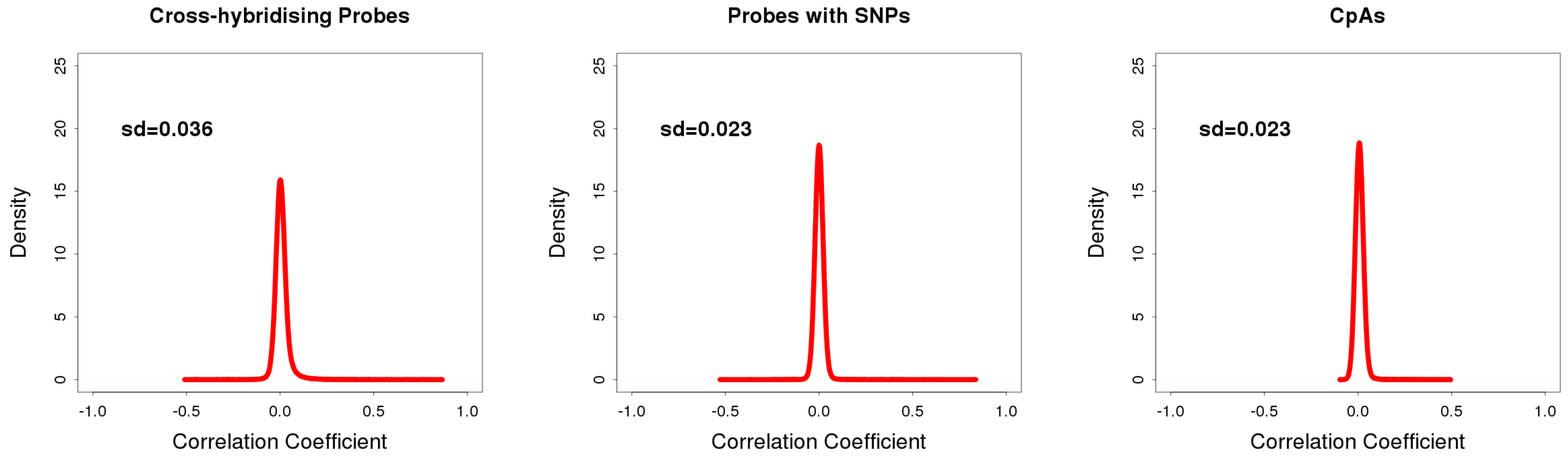

Supplementary Figure 25B. Test statistic distribution under the null hypothesis by permutation testing, for three different marker categories. Beta-values were quantile normalised and adjusted for control probe PCs, age, gender, white-blood cells and PC1-5.

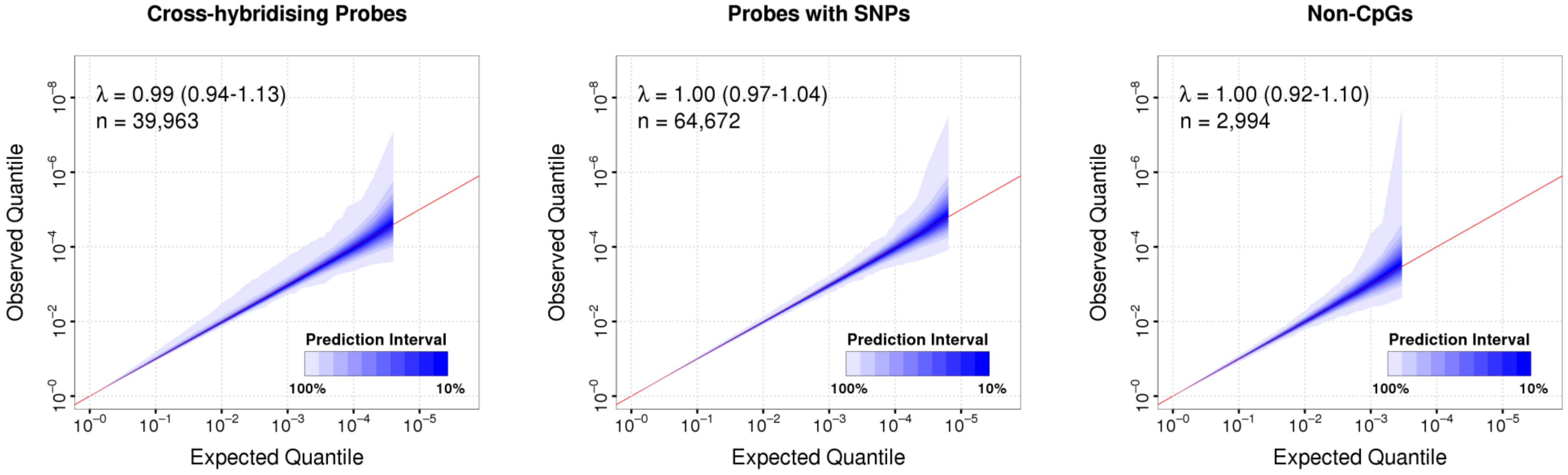

Supplementary Figure 26A. Correlation between 1,000 randomly chosen markers (416 for chromosome Y). We perform a linear regression predicting the beta-value (QN) as a function of control probe PCs, age, white-blood cells and PC1-5 and calculate Pearson correlation coefficients between marker pairs based on the residuals.

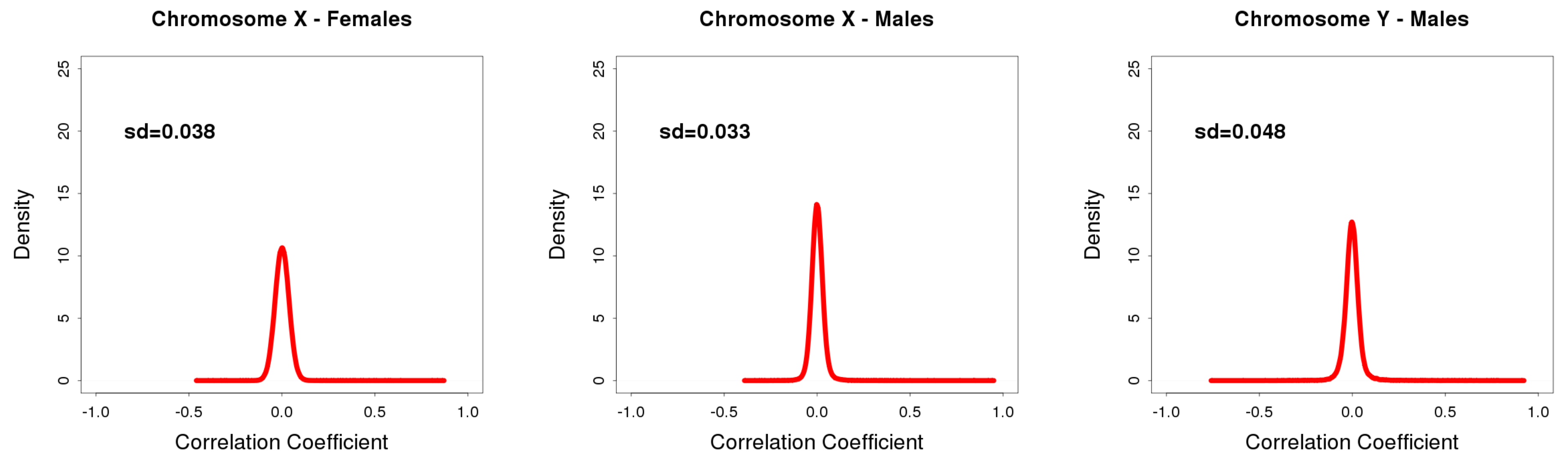

Supplementary Figure 26B. Test statistic distribution under the null hypothesis by permutation testing, for chromosome X and Y. Beta-values were quantile normalised and adjusted for control probe PCs, age, white-blood cells and PC1-5.

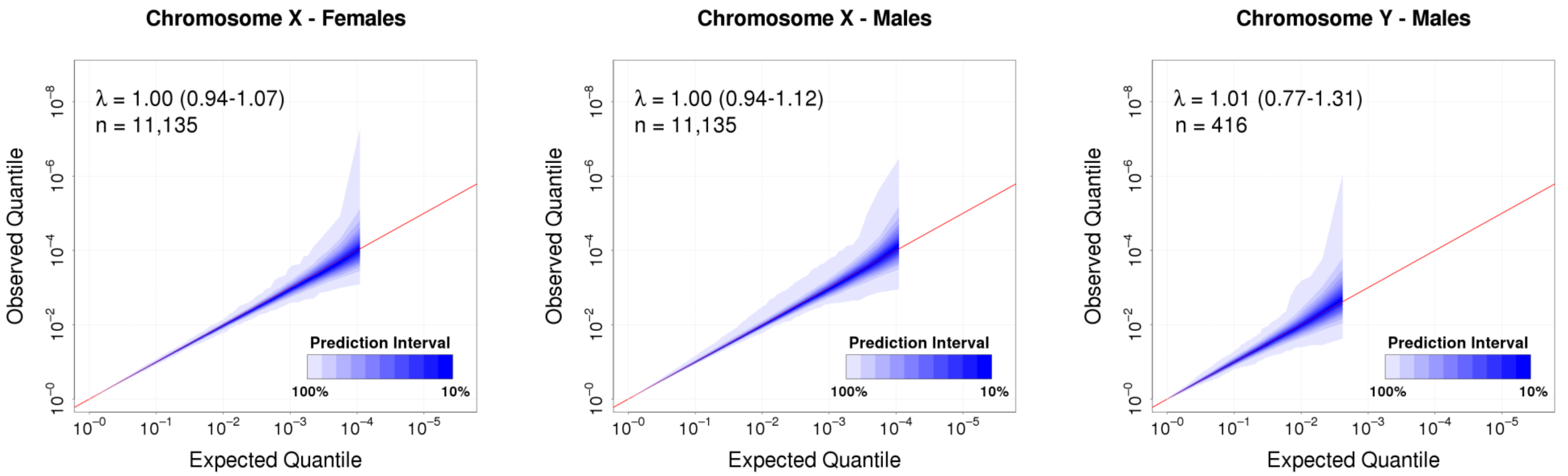

Supplement: Additional file 1: — Contains all supplementary figures and tables. [file 13059_2015_600_MOESM1_ESM.pdf]
